# Gut enterotype- and body mass index (BMI)-dependent effects of anthocyanin supplementation on gut microbiota composition in individuals at risk for cognitive decline: a randomized placebo-controlled trial

**DOI:** 10.1080/19490976.2025.2570862

**Published:** 2025-10-29

**Authors:** Yohannes Seyoum, Chiara de Lucia, Khadija Khalifa, Anne Katrine Bergland, Dag Aarsland, Mark van der Giezen

**Affiliations:** a Department of Chemistry, Bioscience, and Environmental Engineering, University of Stavanger (UiS), Stavanger, Norway; b Centre for Age-Related Medicine (SESAM), Stavanger University Hospital, Stavanger, Norway; c Centre for Healthy Brain Ageing, Department of Psychological Medicine, Institute of Psychiatry, Psychology and Neuroscience (IoPPN), King's College London, London, UK; d Department of Clinical Medicine, University of Bergen, Bergen, Norway; e Biosciences University of Exeter, Exeter, UK; f Research Department, Stavanger University Hospital (SUS), Stavanger, Norway; g Current address: Natural Resources Institute, University of Greenwich, Central Avenue, Chatham Maritime, Kent, UK

**Keywords:** Anthocyanins, enterotype, microbiota, mild cognitive impairment, personalized nutrition, body mass index

## Abstract

Anthocyanins, bioactive flavonoids found in berries, modulate gut microbiota composition and influence health outcomes. This study investigated the effects of anthocyanin supplementation on gut microbiota and cognition in older adults (60–80 y) at risk of cognitive decline due to mild cognitive impairment (MCI) or cardiometabolic disorders (CMD). In a 24-week, randomised, double-blind, placebo-controlled trial (*n* = 99), participants received anthocyanin capsules or placebo. Gut microbiota composition was profiled using 16S rRNA sequencing, considering factors such as baseline enterotype, body mass index (BMI), and age. Overall, alpha diversity remained unchanged, while beta diversity indicated modest but significant intervention effects at the amplicon sequence variants level. Baseline enterotype strongly influenced responsiveness: enterotype one (higher diversity, eubiotic taxa) showed modest but consistent shifts, whereas enterotype two (dysbiotic, Bact2-like) exhibited broader but less coherent changes. BMI-specific responses included enrichment of *Oscillibacter* and *Ezakiella* in healthy-weight individuals and Bacteroidota taxa in obese participants, alongside consistent reductions in Firmicutes. Age stratification revealed heterogeneous, quartile-specific taxa modulations. Cognitive performance, measured by episodic memory, was unaffected, and microbial shifts did not mediate intervention effects. These findings demonstrate that anthocyanins selectively modulate the gut microbiome in an age-, BMI-, and enterotype-dependent manner, underscoring the importance of personalized microbiome-informed nutritional interventions.

## 1. Introduction

Anthocyanins are a subclass of flavonoids responsible for colour in some fruits and vegetables.^1^ Common sources include berries such as blueberries, strawberries, and blackberries, as well as red cabbage and eggplant.^2^ These compounds exhibit diverse bioactive properties, such as reducing oxidative stress, exhibiting antimicrobial activity, and mitigating the progression of diseases including neurodegenerative, cardiovascular, and metabolic disorders, as well as certain cancers.^1^

Despite their potential health benefits, the bioavailability of anthocyanins is limited. After ingestion, only a small proportion is absorbed in the stomach, with absorption rates varying by chemical structure and molecular weight.^2^ This variation in absorption is primarily due to differences in the glycosidic moieties attached to the anthocyanin molecules.^2^ For instance, monoglucoside anthocyanins can achieve absorption rates of up to 25%, whereas other forms exhibit much lower rates (around 1%).^3^ In the small intestine, an additional 5% of dietary anthocyanins are absorbed through passive diffusion or active transport via membrane transporters.^4^ The majority, however, remain unabsorbed and reach the large intestine, where they interact with the gut microbiota.^5^^,^^6^

The human gut microbiome is a complex system consisting of bacteria, archaea, fungi, microbial eukaryotes, and phages, and plays a pivotal role in health and homeostasis.^7^ It aids in digestion and energy extraction,^8^ maintains epithelial barrier function,^9^ supports immune system development,^10^ synthesises vitamins,^11^ and even regulates stress responses.^12^ Among these functions, bacteria are particularly crucial, as they metabolise unabsorbed anthocyanins into bioactive compounds. Microbial enzymes such as *β*-glucosidase, *α*-galactosidase, and *α*-rhamnosidase catalyze the breakdown of anthocyanins into aglycones, which are subsequently converted into phenolic acids like protocatechuic acid, gallic acid, and *p*-coumaric acid. Specific bacterial species, including *Bacteroides* spp*.*, *Enterococcus casseliflavus*, *Eubacterium* spp*.*, and *Clostridium spp.*, facilitate these transformations.^13^

The resulting metabolites not only promote beneficial bacterial populations (e.g., *Bifidobacterium* and *Lactobacillus*) but also suppress harmful species (e.g., *Clostridium histolyticum*).^4^ For example, studies have shown that blackcurrant anthocyanins enhance populations of *Bifidobacterium* and *Lactobacillus* while reducing *Clostridium* and *Bacteroides.*^14^ Similarly, anthocyanins from black rice have been shown to restore short-chain fatty acid (SCFA) levels and beneficial bacteria (*Bifidobacterium spp.* and *Lactobacillus spp.*) in animal models.^15^ A six-week intervention with blueberries increased gut microbial diversity in older women but not in younger women, indicating that age and gender influence the effects of anthocyanins on the gut microbiome.^16^

This bidirectional relationship between anthocyanins and the gut microbiome is characterised by two key interactions: first, anthocyanins can directly influence the composition and function of the gut microbiome by promoting beneficial bacteria and suppressing harmful ones. This modulation can lead to changes in the gut's physiological functions, such as SCFA production,^17^^,^^18^ fat metabolism,^19^^,^^20^ gut barrier integrity,^21^ and gut-brain communication.^22^ Second, the gut microbiome modulates the metabolism and bioactivity of anthocyanins, transforming them into various metabolites, which may have greater stability and bioactivity than the original anthocyanins.^23^ However, this interplay is complex and shaped by various factors, including the baseline microbiome composition, age, gender, and diet. This inherent inter-individual variability in gut microbiome composition and function often complicates broad conclusions from dietary interventions, as it can lead to diverse responses to anthocyanin consumption.^24^^,^^25^ Such variability frequently manifests as distinct community structures, known as enterotypes,^26^ which can profoundly influence an individual's metabolic profile and their unique response to dietary interventions. Furthermore, the existence of polyphenol gut metabotypes, which are subgroups of individuals with similar metabolic profiles, also suggests the dietary interventions must be tailored and personalized.^27^^,^^28^ Current gaps in understanding include how the baseline microbiome influences responses to anthocyanin supplementation, the differences in anthocyanin metabolism between healthy and obese individuals, the long-term effects on microbiome stability, and identifying predictors of individual response based on factors like body mass index (BMI) and age. To address these gaps, this study investigated the effects of anthocyanin supplementation (capsules containing naturally purified anthocyanins from bilberry (*Vaccinium myrtillus*) and black currant (*Ribes nigrum*)) on microbiome composition over a 24-week randomized, double-blind, placebo-controlled Phase II trial in a Norwegian cohort. Specifically, it investigated how baseline microbiome composition, along with intrinsic factors like BMI and age, mediate the microbiome’s response to anthocyanin supplementation, aiming to provide insights for more effective, personalized dietary interventions. In addition, because anthocyanins are proposed to exert neuroprotective effects through the gut–brain axis, we also examined whether microbiome changes were associated with cognitive performance, with a focus on episodic memory as a clinically relevant outcome.

## 2. Methods

### 2.1. Study design and participants

A 24-week randomised, double-blind, placebo-controlled Phase II trial involving 206 participants was conducted between 2018 and 2020 in three cities in Norway: Stavanger, Oslo, and Bergen. The study was reviewed and approved by The Norwegian Regional Ethics Committee (2017/374) and registered with ClinicalTrials.gov (identifier NCT03419039). All participants provided written informed consent.^29^^,^^30^ Eligible participants were aged 60 to 80 y and either had mild cognitive impairment (MCI) diagnosed according to the Winblad criteria,^31^ with or without cardiometabolic disorders (CMD), or were cognitively healthy individuals with at least two CMDs known to increase the risk of cognitive decline and dementia.^29^

Exclusion criteria included a diagnosis of dementia, Parkinson's disease, stroke within the past 5 y, or any other somatic conditions that, according to the study physician, could negatively affect cognitive function. Other exclusions were clinically significant depression, use of anticoagulants, prior use of the investigational product within 12 months, and difficulties using computerized tests. Full details on the inclusion–exclusion criteria, recruitment processes, randomization, data collection, and clinical outcome measurements are available in the primary study publication.^29^

A subsample of 99 participants was selected from the main trial for microbiome analysis, consisting of 45 participants from the placebo group and 54 participants from the intervention group. Fecal samples were collected at screening (baseline), week 12, and week 24. The intervention consisted of two Medox capsules twice a day (Madpalette AS, Sandnes, Norway), each containing 80 mg of purified anthocyanins derived from bilberry (*Vaccinium myrtillus*) and black currant (*Ribes nigrum*) i.e. 320 mg anthocyanins per day. Identically packaged placebo capsules were provided by the same manufacturer.^29^

### 2.2. 16S rRNA sequence analysis

DNA was extracted from stool samples using routine procedures and sent for 16S rRNA sequencing. A V3-V4 region of the 16S rRNA gene was amplified from 629 samples, including technical duplicates, negative and positive controls, using primers 341F (5'-CCTACGGGNGGCWGCAG−3') and 805 R (5'-GACTACHVGGGTATCTAATCC−3').^32^ Amplicons were sequenced on the Illumina MiSeq platform (2 × 300 bp), generating about 40 million raw reads in total. Demultiplexing was performed with Illumina bcl2fastq v2.20, allowing up to two mismatches or ambiguous bases (Ns) in the barcode read if the minimum Hamming distance between barcodes permitted. Adapter trimming was done using BBMerge (v34.48),^33^ and reads shorter than 100 bp after trimming were discarded. Primer sequences were removed allowing up to three mismatches per primer. Quality control was assessed using FastQC (v0.12.1).^34^

Reads were quality trimmed using DADA2 (V1.18).^35^ Forward and reverse reads were truncated at 266 and 261 bp, respectively, to keep bases with a minimum Phred score (Q-score) of 30. Additional filtering parameters included maxEE = 2 (maximum expected errors), truncQ = 2 (truncates reads at the first instance of a quality score ≤ 2), maxN = 0 (discards reads with ambiguous bases), pool = “pseudo” to improve detection of rare variants. Chimeric sequences were removed using the consensus method.

Amplicon sequence variants (ASVs) were assigned taxonomy using a Naïve Bayes classifier via q2-feature-classifier plugin^36^ in QIIME2 (v2023.5.0),^37^ trained on the SILVA 138.1 database^38^ (trimmed to the 341F - 805R region). Sequences that could not be classified at the phylum level or identified as mitochondrial or chloroplast origin were removed.

A phylogenetic tree was generated using MAFFT algorithm for multiple sequence alignment,^39^ followed by FastTree^40^^,^^41^ within QIIME2 using the qiime phylogeny align-to-tree-mafft-fasttree pipeline.^37^ After these preprocessing steps, 13,066,594 quality-filtered reads were retained, with an average of 21,705 reads per sample, and 6526 unique ASVs. To identify and exclude potential contaminants, the Decontam R package (v.1.22.0)^42^ was used, employing the prevalence method with a threshold of 0.5, based on 20 negative controls.). A total of 34 ASVs were flagged as contaminants and removed (Supplementary table S1).

Given that DNA extraction was performed in duplicate and sequencing was randomized across runs, the technical replicate with the highest read depth per sample was selected for downstream analysis. Taxa from rare phyla Elusimicrobiota (mean and total prevalence = 3) and Spirochaetota (mean prevalence = 4.3 and total prevalence = 13) were excluded. ASVs with fewer than 10 total reads across all samples were filtered out.

After these steps, 7,841,478 reads remained, with a range of 7044 to 157,416 reads per sample (median = 25,852), representing 5319 ASVs across 324 unique genera. For diversity analyses, data were rarefied to 12,358 reads per sample, based on the rarefaction curve plateauing (Supplementary figure S1). For differential abundance analysis, only ASVs present in at least 5% of samples were retained.

#### 2.2.1. Gut microbial community typing

Gut microbial community types were identified using the Dirichlet Multinomial Mixture (DMM) model applied to genus-level rarefied count data.^43^ Two optimal clusters were identified based on the Bayesian Information Criterion (BIC), while Laplace’s approximation and Akaike Information Criterion (AIC) suggesting three clusters (supplementary figure S2A-C). We opted for two optimal cluster model based on BIC, which, with its stronger penalization for model complexity, minimizes overfitting,^43^ particularly given the limited representation of a potential third enterotype (supplementary figure S2-D). Comparison of the two clustering methods shows that enterotype one (BIC) has a significant overlap with enterotype one (AIC and Laplace's approximation), while also including samples from enterotype two of the latter (Supplementary Figure 2-D). In contrast, enterotype two (BIC) splits between enterotype two and enterotype three under the three-cluster model, indicating finer resolution within that subgroup. Therefore, selecting two clusters over three enhances both interpretability and robustness, ensuring that the identified groups represent stable and biologically meaningful microbial configurations. Fisher's exact test was used to determine associations between gut community types and categorical metadata variables (*P *< 0.05). Association of BMI and age with gut community types were tested using the Kruskal-Wallis test (*P *< 0.05). The effect of treatment type and time on the distribution of gut microbial community types were analysed using a Generalized Estimating Equations (GEE) approach.^44^ The model (enterotype~treatment type+treatment type×time) used a binomial family with a logit link function and an exchangeable correlation structure.

#### 2.2.2. Statistical analysis

Alpha diversity metrics such as observed richness, Shannon index, and Pielou's evenness were calculated using the microbiome R package (version 1.24.0). Faith's phylogenetic diversity (PD) was calculated using the phyloseq.extended R package (version 0.1.4.1). Beta diversity was calculated using phyloseq R package (version 1.52.0).^45^

Alpha diversity metrics were compared across groups at the baseline using the Kruskal-Wallis test, with multiple comparisons performed using Dunn's post hoc tests. *P* values were adjusted for multiple comparisons using the Benjamini-Hochberg (BH) procedure (*P *< 0.05). Differences in beta-diversity were evaluated using Permutational Multivariate Analysis of Variance (PERMANOVA) with 999 permutations in the vegan R package (version 2.6−4).^46^ Differentially abundant taxa at the baseline across different variables were identified using the Wilcoxon signed-rank test to evaluate differences in microbial abundance distributions.

We assessed the overall effect of the intervention and potential effect modifiers (baseline enterotype, BMI category, and age quartile) on gut microbiome outcomes using baseline-adjusted analysis of covariance (ANCOVA) models for both alpha diversity metrics and genus-level centered-log (CLR)-transformed abundances. For the overall effect, models included treatment group and baseline value as predictors (Week 24 (outcome) ~ treatment type + baseline (genus abundance/alpha diversity)). For stratified analyses, models included an interaction term with the moderator (week 24 (outcome) ~ treatment type × moderator (baseline enterotype/BMI and age quartile) + baseline (genus abundance/alpha diversity). Post hoc pairwise contrasts comparing intervention and placebo within each subgroup were estimated using estimated marginal means (EMMS) and expressed as adjusted mean differences with standard errors. Beta-diversity differences were assessed using repeated measure PERMANOVA using 999 permutations. Models included treatment, time interaction with subject as a stratum to account for repeated measures. Analyses were repeated within the moderators to explore effect modification. Effect sizes are reported as R^2^ with permutation-based *P* values.

Cognitive performance was measured using CogTrack®, a validated online test battery comprising 10 subsets grouped into attention, memory, and cognitive speed domains.^30^ The primary outcome was Quality of Episodic Memory (QEM), a composite derived from four accuracy-based tasks: immediate and delayed word recall, word recognition, and picture recognition. Missing baseline values were imputed using stratified mean imputation, stratified by center, diagnosis and CMD or MCI, see for the details^30^). Intervention effects on QEM at week 24 were evaluated using ANCOVA with covariates (age and gender). Effect modification by baseline enterotype, BMI category, and age quartile was tested by additional interaction terms (treatment × moderator). EMMs were computed for intervention vs placebo within each subgroup.

To explore microbiome-cognition associations, we computed change scores for genus level abundances (∆CLR = week 24 − baseline) and modeled their interaction with QEM at week 24 using ANCOVA (QEM (week−24) ~ (∆CLR + treatment + baseline QEM + gender + age quartile + BMI + baseline enterotype). Stratified analyses incorporating three-way interactions ((∆CLR × treatment type × stratum) for baseline enterotype, BMI and age quartile. Mediation analysis was performed using the mediate R package, estimating average causal mediation effect (ACME), average direct effect (ADE), and total effect via 1000 bootstrap simulations, with ∆CLR as the mediator. *P* values were adjusted for multiple testing using Benjamini-Hochberg (BH) method (*P *< 0.05).

## 3. Results

### 3.1. Participant characteristics

Ninety-five percent of the participants in both groups adhered to their assigned intervention.^29^ The proportion of women in the treatment groups ranged from 49 - 53% (supplementary table S2). The age of participants ranged from 60 to 80 y old. Age distribution (quartile) between intervention groups was significantly different (Chi-square (χ^2^), *P* = 0.007). In the intervention group, 42% were aged 65–68 y, compared to 17% in the placebo group. On the contrary, 31% of the placebo group and 11% of the intervention group were aged 68–73 y. Additionally, approximately 44%–51% of the participants were overweight (BMI, 25–29.9), with 22%–37% classified as obese (BMI ≥ 30). The distribution of the different BMI groups (healthy weight, overweight, and obese) did not differ significantly between the treatment groups (*P *> 0.05). About 75% of the participants had cardiometabolic diseases (CMD), while the remaining participants had mild cognitive impairment (MCI). Accordingly, the proportion of overweight (52.7%) and obese (33.8%) individuals was higher in the CMD group compared with the MCI group (Chi-square (χ^2^), *P* = 0.001). The distribution of CMD and MCI were similar across the treatment groups. Additionally, the percentages of participants who were smokers, consumed alcohol, and had coronary heart disease, diabetes, familial dementia, hypertension, or hypercholesterolemia were similar between groups. The use of medications such as diabetes treatments, calcium channel blockers, anti-inflammatory or antirheumatics, thyroid therapies, and proton pump inhibitors was also similar across the groups.

### 3.2. Baseline gut microbiome composition and variation by enterotype, BMI, and age

#### 3.2.1. Baseline microbiome composition

**Figure 1.**
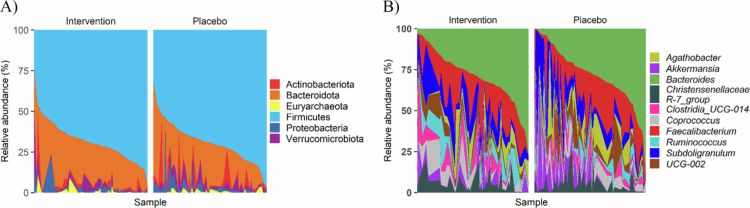
Relative abundance (%) of the top six phyla and top ten genera, partitioned by treatment group at baseline. Each bar represents an individual sample, with colors indicating specific phyla or genera. A) Phylum-level composition, B) top ten genera composition.

A total of 5319 ASVs were identified, comprising 12 phyla (10 bacterial and 2 archaeal) and 324 unique genera. The baseline microbiota was dominated by Firmicutes (69.8%), followed by Bacteroidota (21.3%) and Actinobacteria (3.08%) (Figure 1A). Euryarchaeota was the least abundant phylum (0.78%), represented solely by *Methanobrevibacter*. At the genus level, *Bacteroides* (15.3%), *Faecalibacterium* (9.2%), *Subdoligranulum* (6.6%), and *Agathobacter* (3.4%) were most abundant (Figure 1B).

We first characterized the baseline gut microbiome composition and enterotype profiles to establish the overall microbiome community structure. We then examined how gut microbial diversity and composition vary with host factors. Among these, BMI was uniquely associated with differences in alpha diversity, while age was the only factor significantly linked to beta diversity. To further understand these associations, we next characterized genus-level differences stratified by BMI and age groups (quartiles).

#### 3.2.2. Gut microbiome community types (enterotypes) characterization at the baseline

**Figure 2.**
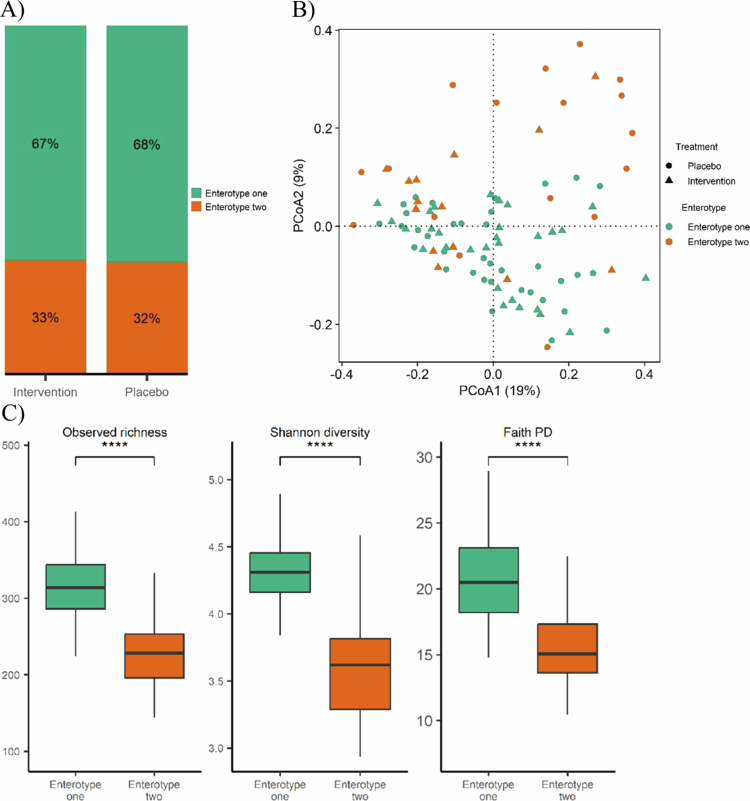
Clustering of gut microbiota communities based on Dirichlet multinomial mixtures (DMM) at the genus level at the baseline. A) Distribution of enterotypes across treatment groups. B) Principal coordinates analysis (PCoA) of Bray-Curtis distance at different treatment and enterotype groups (PERMANOVA, *P* = 0.002, *R*^*2*^ = 0.044). C) Alpha diversity indices (observed richness, Shannon index and Faith's phylogenetic diversity (PD)) per each enterotype group. Differences in alpha diversity measures between enterotypes were assessed using the Wilcoxon signed-rank test. ****P < 0.001.

DMM modeling identified two enterotypes across all time points according to BIC criterion (Supplementary figure S2C). They represent a group of metacommunities harbouring similar microbial configurations. At baseline, 68% and 32% of the participants belonged to enterotype one and two, respectively. Distribution of enterotypes did not differ by treatment group (Figure 2A) and was not associated with lifestyle factors, health status, demographic variables, or medication use (Fisher’s exact test, BH adjusted *P *> 0.05).

Beta diversity analysis using Bray-Curtis dissimilarity revealed a significant difference between enterotype groups (*P* = 0.002, Figure 2B), indicating that about 4.4% of the interindividual variation in microbiome structure could be attributed to enterotype. Enterotype one was characterized by high observed richness (*P *< 0.001), Shannon diversity (*P *< 0.001), and phylogenetic diversity (Faith's PD, *P *< 0.001, Figure 2C). Both enterotypes were dominated by Bacteroides, but enterotype one harbored significantly higher levels of anti-inflammatory taxa such as *Faecalibacterium* (9.8% vs. 7.8%, BH-adjusted *P* = 0.04), short-chain fatty acid producers including *Coprococcus* (3.3% vs. 2.0%, *P* = 0.01) and *Alistipes* (3.4% vs. 1.4%, *P* = 0.002), fibre fermenters such as *Clostridia UCG−014* (3.1% vs. 2.5%, *P* = 0.007), and taxa associated with low BMI, such as *Christensenellaceae R−7 group* (3.3% vs. 1.6%, *P* = 0.0007) and *Methanobrevibacter* (0.9% vs. 0.5%, *P* = 0.003; supplementary table S3).

Enterotype two, by contrast, displayed lower alpha diversity and significantly higher levels of *Eggerthella* (BH adjusted *P* = 0.004),a genus considered part of a normal microbiota but also linked to gastrointestinal infections as well as bacteraemia.^47^ *Ruminococcus gnavus group* was also more abundant (0.04% vs. 1.2%, BH-adjusted *P* = 0.03). Several other genera were differentially abundant between the two enterotypes (supplementary table S3). Enterotype two closely resembled the dysbiotic “Bact2” enterotype reported elsewhere.^48^^,^^49^ Neither treatment nor time changed the enterotype distribution, as determined by a multinomial logistic regression model. Given the differences in diversity and taxa between the enterotypes, they will likely have different metabolic profiles and gut microbiome responses under the same diet conditions. To investigate this, we examined whether baseline enterotype stratification could influence the microbiome's response to anthocyanin supplementation.

#### 3.2.3. BMI-associated microbiome variation at baseline

**Figure 3.**
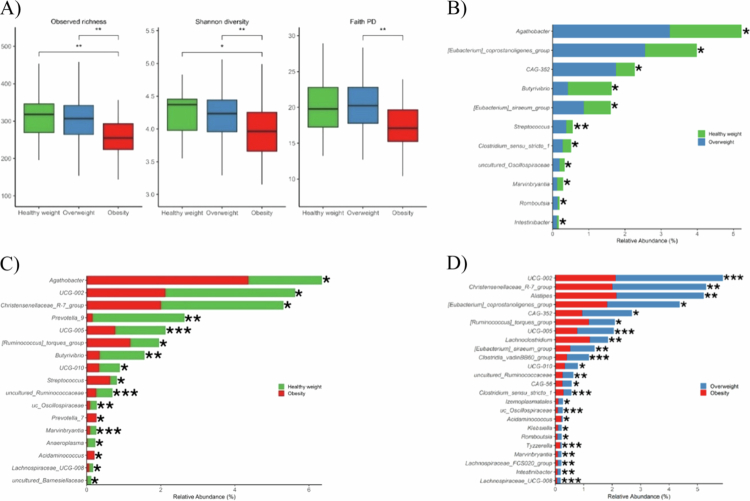
Alpha diversity and mean relative abundance (%) of differentially abundant taxa at the genus level across BMI (body mass index) groups. A) alpha diversity in body mass index (BMI) groups, B) healthy weight compared with overweight, C) healthy weight compared with obesity, D) overweight compared with obesity. Wilcoxon signed-rank test was used to test significance. Only genera with a relative abundance of ≥0.1% in one of the groups being compared are presented. *P* values were adjusted using Benjamini-Hochberg method (*P *< 0.05). The significance of the results is denoted as follows: ****P < 0.0001, ***P < 0.001, **P < 0.01, and * *P* < 0.05.

BMI was significantly associated with gut microbiome diversity at baseline. Obese individuals exhibited significantly lower observed richness and Shannon diversity compared with both healthy weight and overweight participants (Figure 3A; Kruskal–Wallis test with Dunn's post hoc test, *P *< 0.05). Faith's PD was also reduced in obesity relative to the overweight group, whereas no significant differences were observed between healthy weight and overweight individuals.

At the genus level, 61 taxa were significantly associated with BMI, primarily belonging to Firmicutes and Bacteroidota (Figure 3B–D). Genera such as *Agathobacter*, *Clostridium sensu stricto 1*, and *Streptococcus* increased with higher BMI, whereas *Christensenellaceae R−7 group*, *UCG−002*, *UCG−005*, and *Victivallis* decreased.

Obese participants had higher abundances of *Agathobacter* (*P* = 0.003), *Acidaminococcus* (*P* = 0.013), and *Holdemania* (*P* = 0.012), and lower abundances of *Coprobacter* (*P* = 4.23 × 10^−5^), *Prevotella 9* (*P* = 0.004), and *Christensenellaceae R−7 group* (*P* = 0.037) compared to healthy weight individuals. Compared with overweight participants, they also had higher abundances of *Acidaminococcus* (*P* = 0.025) and *Holdemania* (*P* = 2.69 × 10^−4^), and lower abundances of *Alistipes* (*P* = 0.003), *Christensenellaceae R−7 group* (*P* = 0.002), and *UCG−002* (*P* = 3.45 × 10^−5^). Non-monotonic patterns were observed, with *Acidaminococcus* and *Holdemania* enriched in obesity but not differing between healthy weight and overweight, whereas *Marvinbryantia* decreased consistently across all comparisons (*P *< 0.05).

#### 3.2.4. Age associated microbiome variation at baseline

**Figure 4.**
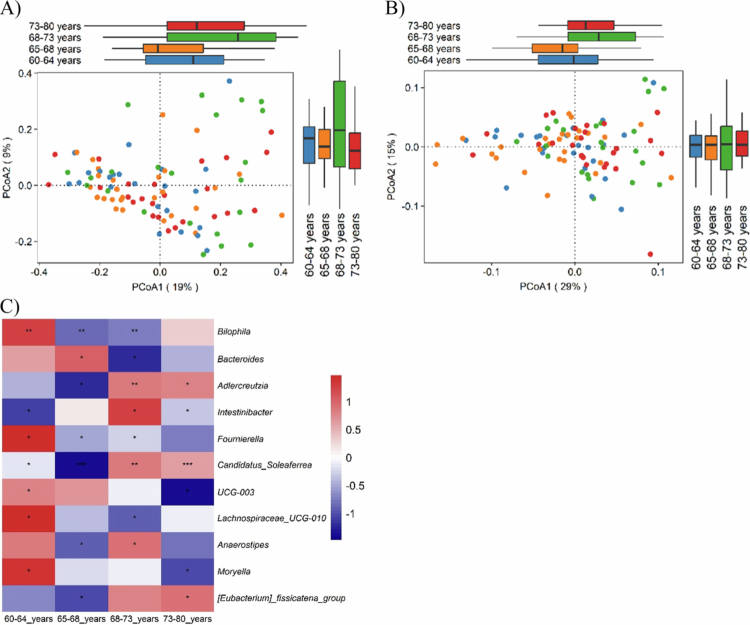
Beta diversity analysis by age (quartiles) and differential abundant taxa at the genus level. A) Principal coordinate analysis (PCoA) of Bray-Curtis dissimilarity at the genus level. B) PCoA of weighted UniFrac distance at the amplicon sequence variant (ASV) level. Permutation analysis of variance (PERMANOVA) was used to compare beta diversity groups (*P* = 0.001). Box plots next to or above the PCoA plots represent PCoA 1 and PCoA 2 axes, respectively. Box plots display the median, and the interquartile range. C) Heatmap of genus relative abundance (%) across age quartiles. Each row represents bacterial genus, and columns correspond to age quartiles (60–64, 65–68, 68–73, 73–80 y). Abundances were row-scaled for visualization. Significance of pairwise differences between age quartiles is indicated as follows: *BH-adjusted *P *< 0.05, **P < 0.01, ***P < 0.001.

Among host factors, only age, stratified into quartiles, was significantly associated with overall community structure. Age explained 4.4% of the variation in genus-level community composition (Bray-Curtis, *P* = 0.04) and 6.7% of the variation in phylogenetic structure (weighted UniFrac, *P* = 0.005) (Figure 4A and B). In contrast, Jaccard and unweighted UniFrac metrics showed no significant associations.

Pairwise PERMANOVA indicated that genus-level community composition differed only between the 65–68 and 68–73 y groups (*P* = 0.01; supplementary table S4). Weighted UniFrac revealed broader differences between 65–68 and 73–80 (*P* = 0.03), 65–68 and 68–73 (*P* = 0.004), and 60–64 and 68–73 (*P* = 0.008) year groups.

Differential abundance analysis identified 13 age-associated genera, 72% of which belonged to Firmicutes (Figure 4C). *Bilophila* and *Fournierella* were enriched in the 60–64 y group compared with both 65–68 (*P* = 0.008 and *P* = 0.023, respectively) and 68–73 y (*P* = 0.008 and 0.045). *Candidatus Soleaferrea* increased progressively with age, with significant differences across consecutive groups (e.g., 65–68 vs. 60–64, *P* = 0.018; 68–73 vs. 65–68, *P* = 0.007; 73–80 vs. 65–68, *P* = 0.0006). *Intestinibacter* was more abundant in 68–73 (*P* = 0.012) and 73–80 (*P* = 0.048) groups compared with 60–64. Additional genera, including *Adlercreutzia* and *Anaerostipes*, increased in older groups, whereas *Bacteroides* was reduced in 68–73 compared with 65–68 y (*P* = 0.023). These findings highlight age-related shifts in gut microbiome composition, with *Candidatus Soleaferrea*, emerges as a potential biomarker of chronological aging in this cohort.

### 3.3. Gut microbiome responses to anthocyanin intervention across enterotype, BMI, and age subgroups

#### 3.3.1. Overall effects of anthocyanin intervention on the gut microbiome

The effect of the intervention on gut microbiome diversity and composition was assessed between the intervention and placebo groups. Alpha diversity and genus-level abundances were analysed using baseline-adjusted linear models (ANCOVA). Beta diversity was evaluated using longitudinal PERMANOVA, stratified by subject to account for repeated measures. No significant differences in any alpha diversity metrics were observed between the intervention and placebo groups at 24 weeks (supplementary table S5, BH adjusted *P *< 0.005). Beta diversity at the genus level showed no significant differences between intervention and placebo groups (Bray-Curtis, R² = 0.013, *P* = 0.061, supplementary table S5). At the ASV level, Bray-Curtis (R² = 0.013, *P* = 0.01) and Jaccard (R² = 0.014, *P* = 0.036) distances showed modest but significant effects, whereas weighted UniFrac was not significant (R² = 0.012, *P* = 0.21) and unweighted UniFrac indicated a small but significant effect (R² = 0.013, *P* = 0.001).

**Figure 5.**
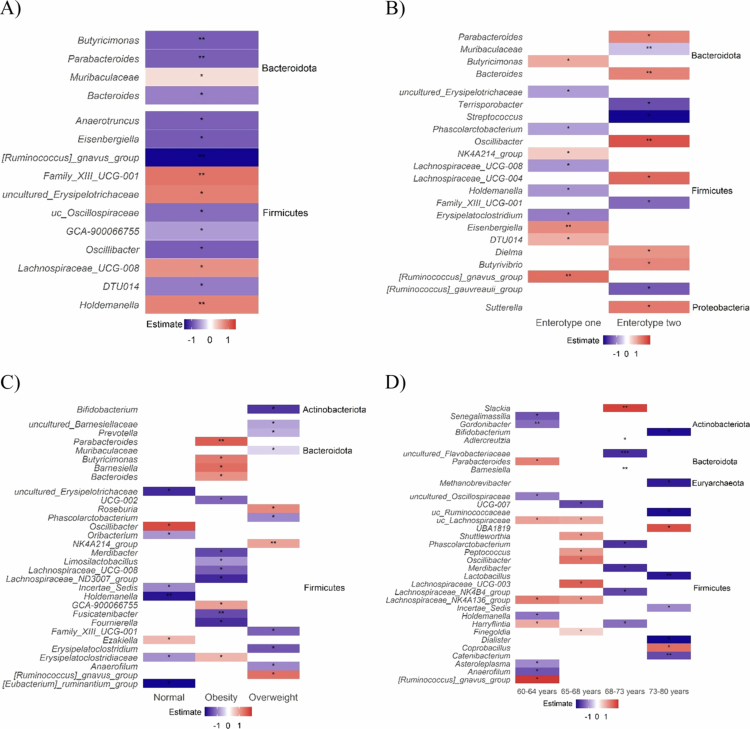
Multi-panel heatmap depicting the effects of anthocyanin supplementation on genus-level gut microbiome composition at week 24. A) Overall intervention effect versus placebo. B) Enterotype-specific effects. C) BMI-specific effects. D) Age-specific effects. Tiles display the adjusted mean difference in centered-log ratio (CLR) abundance between intervention and placebo groups. Red tiles indicate genera increased under intervention; blue tiles indicate decreased abundance. Only genera with Benjamini–Hochberg adjusted *P *< 0.05 are shown. Phylum-level classification is indicated on the right of each panel. Significance is denoted as follows: *P *< 0.05 (*), *P *< 0.01 (**), *P *< 0.001 (***).

Baseline-adjusted ANCOVA revealed that the intervention was associated with significant changes in multiple gut bacterial genera at week 24 (Figure 5A). Increases were observed in *Holdemanella* (estimate = 0.84, *P* = 0.006), *Family XIII UCG−001* (estimate = 0.95, *P* = 0.010), uncultured genus in *Erysipelotrichaceae* (estimate = 0.87, *P* = 0.017), *Lachnospiraceae UCG−008* (estimate = 0.75, *P* = 0.025), and *Muribaculaceae* (estimate = 0.23, *P* = 0.040). Reductions were detected in *Ruminococcus gnavus group* (estimate = −1.43, *P* = 0.002), *Parabacteroides* (estimate = −0.88, *P* = 0.005), *Butyricimonas* (estimate = −0.89, *P* = 0.006), *Bacteroides* (estimate = −0.69, *P* = 0.012), *Anaerotruncus* (estimate = −0.87, *P* = 0.026), *Eisenbergiella* (estimate = −0.90, *P* = 0.031), *Oscillibacter* (estimate = −0.89, *P* = 0.031), *DTU014* (estimate = −0.70, *P* = 0.039), unclassified genus in *Oscillospiraceae* (estimate = −0.79, *P* = 0.041), and *GCA-900066755* (estimate = −0.53, *P* = 0.041). These findings indicate that the intervention selectively modulated gut bacterial composition after accounting for baseline abundance, with significant shifts observed in multiple Firmicutes and Bacteroidota genera.

#### 3.3.2. Enterotype specific microbiome responses to intervention

Similarly, alpha diversity metrics did not differ significantly between intervention and placebo groups within either enterotype at 24 weeks (supplementary table S6). Beta diversity analyses revealed clear enterotype-dependent effects (supplementary table S6). At the genus level, Bray-Curtis dissimilarity showed no significant separation in enterotype one, whereas enterotype two exhibited significant divergence between groups (R² = 0.06, *P* = 0.006). At the ASV level, enterotype one remained unchanged (R² = 0.02, *P* = 0.07), while enterotype two showed significant differences (R² = 0.05, *P* = 0.02). Weighted UniFrac reflected significant intervention-associated shifts in enterotype two (R² = 0.045, *P* = 0.014), but not in enterotype one (R² = 0.02, *P* = 0.21). Unweighted UniFrac revealed significant changes in both enterotypes, with stronger effects in enterotype two (E1: R² = 0.02, *P* = 0.006; E2: R² = 0.045, *P* = 0.003). Jaccard distances did not show significant differences in either enterotype.

In enterotype one, the intervention selectively modulated a limited set of taxa (Figure 5B). *Ruminococcus gnavus group* (estimate = 1.66, *P* = 0.002), *Eisenbergiella* (estimate = 1.33, *P* = 0.009), and *Butyricimonas* (estimate = 0.95, *P* = 0.020) were enriched, while *Erysipelatoclostridium* (estimate = −1.18, *P* = 0.030), *Holdemanella* (estimate = −0.95, *P* = 0.010), and Lachnospiraceae UCG−008 (estimate = −0.95, *P* = 0.020) were reduced. Additional taxa including *Phascolarctobacterium*, DTU014, NK4A214 group, and an uncultured genus in *Erysipelotrichaceae* showed smaller, consistent effects. Overall, responses in enterotype one were modest and predominantly confined to Firmicutes, reflecting limited microbiome plasticity.

In enterotype two, the intervention elicited broader compositional changes. Enriched genera included *Oscillibacter* (estimate = 1.98, *P* = 0.008), *Lachnospiraceae UCG-004* (estimate = 1.73, *P* = 0.048), *Sutterella* (estimate = 1.54, *P* = 0.035), and *Bacteroides* (estimate = 1.41, *P* = 0.005). Conversely, significant reductions were observed in *Streptococcus* (estimate = −2.39, *P* = 0.015), *Ruminococcus gauvreauii group* (estimate = −1.52, *P* = 0.017), *Terrisporobacter* (estimate = −1.64, *P* = 0.021), *Family XIII UCG−001* (estimate = −1.37, *P* = 0.026), and *Muribaculaceae* (estimate = −0.56, *P* = 0.006). These results indicate that enterotype two harbors a broader set of responsive taxa spanning Firmicutes, Bacteroidota, and Proteobacteria, underscoring enterotype-dependent intervention effects.

These findings underscore the complexity of enterotype-specific microbial responses to intervention, highlighting the potential for baseline enterotype classification to guide microbiome-targeted therapeutic strategies. The enterotype-dependent shifts in microbial composition observed here support a personalized approach in microbiome research and therapy, recognizing the significant influence of baseline microbial profiles on the outcomes of dietary or therapeutic interventions.

#### 3.3.3. BMI dependent microbiome responses to intervention

We next examined whether intervention elicited baseline BMI-specific microbiome responses. The intervention did not affect any of the alpha diversity in BMI dependent manner (supplementary table S7). Beta-diversity analyses revealed no significant differences across groups, with the exception of unweighted UniFrac (R^2^ = 0.043, *P* = 0.02), indicating presence–absence-based changes at the phylogenetic level.

At the genus level, however, several genera were modulated in a BMI-specific manner (Figure 5C). In participants with healthy weight, the intervention was associated with a significant increase in *Oscillibacter* (estimate = 1.81, *P* = 0.038) and *Ezakiella* (estimate = 0.72, *P* = 0.023). In contrast, several Firmicutes genera, including *Oribacterium* (estimate = −0.83, *P* = 0.037), *Holdemanella* (estimate = −1.94, *P* = 0.003), and members of *Erysipelotrichaceae* (estimate = −0.87 to −1.77, *P *< 0.05), were significantly reduced.

Among overweight participants, intervention increased abundances of the *Ruminococcus gnavus group* (estimate = 1.43, *P* = 0.034), *Roseburia* (estimate = 1.16, *P* = 0.030), and *NK4A214 group* (estimate = 0.92, *P* = 0.007). In contrast, reductions were observed in multiple *Bacteroidota* genera (Muribaculaceae, −0.33, *P* = 0.04; *Prevotella*, −0.62, *P* = 0.041; *Barnesiellaceae*, −0.71, *P* = 0.034), as well as Firmicutes including *Phascolarctobacterium* (−0.93, *P* = 0.035) and *Anaerofilum* (estimate = −0.94, *P* = 0.040).

In the obese participants, intervention was consistently associated with increases in several *Bacteroidota* taxa (*Parabacteroides*, 1.63, *P* = 0.006; *Barnesiella*, 1.46, *P* = 0.020; *Butyricimonas*, 1.32, *P* = 0.030; *Bacteroides*, 1.08, *P* = 0.040). Depletion was observed in multiple Firmicutes genera including *Limosilactobacillus* (−0.84, *P* = 0.030), *Fusicatenibacter* (−1.45, *P* = 0.002), *Merdibacter* (−1.49, *P* = 0.030), and *Lachnospiraceae ND3007 group* (estimate = −1.78, *P* = 0.020). These findings demonstrate that the intervention induced BMI-specific shifts, with enrichment of distinct taxa in each BMI category and consistent depletion of Firmicutes members, particularly *Erysipelotrichaceae* and *Lachnospiraceae*, across overweight and obese participants. Overall, these results underscore the potential of anthocyanin intervention to foster a healthier gut microbiome across BMI categories, with implications for metabolic health. The intervention supported an increase in beneficial primary fermenter bacteria and reduced the prevalence of taxa linked to metabolic disorders, suggesting a targeted approach that could help mitigate chronic disease risk by promoting a stable and beneficial gut microbiota.

#### 3.3.4. Age dependent microbiome response to intervention

Given the influence of age (quartile) on baseline microbiome structure, as reflected in distinct beta diversity profiles, we investigated whether age also shaped the microbiome's response to intervention. ANCOVA contrasts stratified by age quartile showed no significant intervention effects on alpha diversity (supplementary table S8). Beta diversity analyses revealed significant intervention-related differences in the 68–73 y group for Bray-Curtis dissimilarity at the genus level (R^2^ = 0.074, *P* = 0.019) and ASV level (R^2^ = 0.065, *P* = 0.038). In addition, unweighted UniFrac distances differed significantly in the 73–80 y group (R^2^ = 0.062, *P* = 0.017). No significant intervention effects were observed for the other diversity metrics across age groups.

Genus-level analyses revealed distinct age-dependent responses (Figure 5D). In the 60–64 y group, intervention was associated with increased abundances of the *Ruminococcus gnavus* group (estimate = 2.29), *Lachnospiraceae NK4A136* group (estimate = 1.49), *Harryflintia* (estimate = 0.95), and *Parabacteroides* (estimate = 1.44), alongside reductions in *Asteroleplasma* (estimate = −1.06), *Holdemanella* (estimate = −1.26), *Gordonibacter* (estimate = −1.33), and *Senegalimassilia* (estimate = −1.57). In the 65–68 y group, enrichment was observed for *Lachnospiraceae UCG−003* (estimate = 1.84), *Oscillibacter* (estimate = 1.62), *Shuttleworthia* (estimate = 1.19), *Peptococcus* (estimate = 1.15), and *Finegoldia* (estimate = 0.52), while *UCG−007* decreased significantly (estimate = −1.71). In the 68–73 y group, *Adlercreutzia* (estimate = 2.46) and *Slackia* (estimate = 2.18) were enriched, whereas *Harryflintia* (estimate = –1.31), *Lachnospiraceae NK4B4 group* (estimate = −1.73), *Phascolarctobacterium* (estimate = −1.94), *Merdibacter* (estimate = −1.99), and *Barnesiella* (estimate = −2.66) were reduced. Finally, in the 73–80 y group, intervention increased abundances of *UBA1819* (estimate = 2.02) and *Coprobacillus* (estimate = 1.65), while markedly reducing *Catenibacterium* (estimate = -1.69), *Dialister* (estimate = −2.38), *Bifidobacterium* (estimate = −2.30), *Methanobrevibacter* (estimate = −2.17), and *Lactobacillus* (estimate = −2.23). Together, these findings indicate that the intervention exerted heterogeneous, age-dependent effects on the gut microbiota, with distinct responsive taxa emerging across different age groups.

### 3.4. Associations between microbiome features and cognitive performances

In addition to microbiome outcomes, we assessed cognitive performance using QEM score as a prespecified clinical endpoint. An ANCOVA model, adjusting for baseline QEM, age quartile, gender (following the approach of the parent study^50^), and baseline BMI, revealed no significant effect of the anthocyanin supplementation on QEM at week−24 compared to placebo (estimate = 2.49, *P* = 0.22, supplementary table S10). Baseline QEM was a robust predictor of endline QEM (estimate = 0.43, *P *< 0.001) reflecting stability in cognitive performance. We explored potential effect modification by baseline enterotype, BMI category, and age quartile, but none of these factors significantly altered the intervention’s impact on QEM (*P *> 0.05). Gender showed a borderline association with QEM in the age-stratified model, suggesting a possible secondary influence. Given the observed direct effect of intervention and modifier-dependent changes in several genera, we next examined whether changes in genus-level CLR-transformed abundances (∆CLR, week 24 − baseline) were associated with QEM. In the unified model adjusting for baseline QEM, age quartile, gender, BMI, and enterotype, we detected ten significant terms across eight genera after *P* value correction.

For some genera, ∆CLR was associated with QEM regardless of intervention: for example, *Phocea* (estimate = −2.16, *P* = 0.016) and *Slackia* (estimate = −2.89, *P* = 0.008) showed overall negative associations, indicating that greater ∆CLR corresponded to lower QEM. In contrast, several effects were specific to the intervention group, captured by the ∆CLR and Intervention interaction term. Notably, *Acetanaerobacterium* (estimate = −6.23, *P* = 0.0011) and *Clostridium sensu stricto*-1 (estimate = −2.37, *P* = 0.020) were associated with reduced QEM in the intervention group, whereas *Slackia* (estimate = 4.58, *P* = 0.0033) showed the opposite pattern, with higher QEM linked to greater ∆CLR in the intervention group. These findings indicate that the relationship between microbial changes and cognition was partly contingent on intervention status. Stratified analyses revealed further subgroup-specific effects. For example, *Barnesiella* was positively associated with QEM in the intervention group (estimate = 17.44, *P* = 0.02), but this association reversed in participants aged 73–80 y (estimate = −27.5, *P* = 0.02). Similarly, *Moryella* showed a positive association in the intervention group (estimate = 17.08, *P* = 0.03) but a negative association in those aged 65–68 y (estimate = −16.52, *P* = 0.03). An uncultured genus in Peptococcaceae family was also negatively associated with QEM in the 65–68 y group (estimate = −7.92, *P* = 0.03). These subgroup-specific patterns highlight that the cognitive impact of microbiome shifts varied not only with intervention status but also across age strata, with positive coefficients indicating higher QEM and negative coefficients indicating lower QEM.

Mediation analysis assessed whether ∆CLR changes mediated the intervention's effect on QEM for the 11 genera with significant unified or stratified model associations. All genera displayed non-significant mediation effects (ACME *P *> 0.05). For instance, *Acetanaerobacterium* yielded an ACME of −0.30 (*P* = 0.658), *Slackia* an ACME of −0.03 (*P* = 0.744), and *Barnesiella* an ACME of −0.03 (*P* = 0.960). Average Direct Effects (ADE) and Total Effects remained non-significant (all *P *> 0.05), with proportion mediated ranging from −0.26 to 0.15 (all *P *> 0.05). These results suggest that, despite correlations between genus abundance changes and QEM, they do not mediate the intervention's lack of effect (*P* = 0.22). Larger samples and longitudinal designs will be needed to test microbiome–cognition mediation more robustly.

## 4. Discussion

This study investigated the effects of purified anthocyanins on gut microbiota composition in older adults (60–80 y) at risk of cognitive decline due to mild cognitive impairment (MCI) or cardiometabolic disorders (CMD). Using a randomized, placebo-controlled design to rigorously evaluate dietary effects on cognitive health,^51^ we found that the response to anthocyanin supplementation was influenced by baseline microbiota composition (enterotype), BMI, and age. These findings highlight the complexity of host-microbiota-diet interactions and emphasize the need for personalized approaches in dietary interventions. Notably, while no direct intervention effect on cognitive performance was observed, changes in specific microbial taxa correlated with cognitive outcomes, suggesting indirect links via the gut-brain axis that warrant further exploration in aging cohorts.

Overall, anthocyanin supplementation did not significantly alter alpha diversity metrics, such as richness and evenness. This is consistent with earlier studies showing that dietary polyphenols primarily affect individual microbial taxa rather than overall diversity. For example, a study investigating the effects of anthocyanin-rich raspberry extract fermented with gut microbiota from various hosts reported increased alpha diversity in human adults and rats, while remaining stable in infants and mice,^52^ underscoring the role of host-specific factors and microbiota source in shaping microbial responses. Beta diversity analyses revealed intervention effects at the ASV level, suggesting community restructuring. A significant increase in *Holdemanella*, *Family XIII UCG−001*, *Lachnospiraceae* members, and *Muribaculaceae* due to intervention was observed, which are associated with short-chain fatty acid (SCFA) production, anti-inflammatory effects, and support metabolic health in older adults.^53^^,^^54^ *Holdemanella,* particularly *Holdemanella biformis*, has been linked to improved glucose tolerance, anti-inflammatory effects, and enhanced gut barrier integrity via SCFA production.^55^ Conversely, reductions were observed in *Ruminococcus gnavus* group, *Parabacteroides*, *Butyricimonas*, and *Oscillibacter*. The *Ruminococcus gnavus* group is often enriched in inflammatory and metabolic disorders, with potential to exacerbate gut barrier disruption.^56^ *Parabacteroides*, while sometimes beneficial in metabolic health, can be contextually dysbiotic in aging cohorts.^57^ *Butyricimonas*, a butyrate producer, shows mixed associations, with reductions potentially reflecting trade-offs in community dynamics.^58^ These shifts suggest anthocyanins foster anti-inflammatory and metabolically favorable gut environments.

The identification of two distinct *Bacteroides-*dominated enterotypes in our cohort*,* rather than four commonly reported previously,^49^^,^^59^ highlights the influence of cohort-specific factors, including diet, lifestyle, and health status, on enterotype classification.^60-62^ Responses were stronger in enterotype two, with broader taxonomic changes spanning Firmicutes and Bacteroidota and significant beta diversity shifts. An enrichment of taxa inversely associated with cardiometabolic risks (*Oscillibacter*), and SCFA producers (*Lachnospiraceae UCG-004*) and reduction of *Streptococcus* were observed as an example. These patterns suggest dysbiotic enterotypes may exhibit greater responsiveness to anthocyanins, potentially due to ecological instability allowing for more extensive remodeling towards eubiosis, with implications for personalized interventions in inflammation-prone older adults.

BMI-dependent responses were evident, with obese individuals showing lower baseline alpha diversity, consistent with established obesity-related dysbiosis patterns characterized by reduced microbial gene richness.^63-65^ The intervention did not change alpha diversity in a BMI-dependent manner but elicited presence–absence shifts in microbial composition. In healthy-weight participants, increases in *Oscillibacter* and *Ezakiella* occurred alongside reductions in *Holdemanella.* Overweight individuals showed enrichments in *Roseburia*, but reductions in *Muribaculaceae* and *Prevotella*. In obese participants, Bacteroidota taxa dominated the increases, including *Parabacteroides*; alleviating obesity via succinate and bile acids, *Barnesiella*, *Butyricimonas*, and *Bacteroides*; enhancing carbohydrate metabolism, with depletions in Firmicutes such as *Fusicatenibacter*. These BMI-specific shifts underscore anthocyanins' potential to counteract obesity-driven dysbiosis by promoting beneficial fermenters and reducing inflammation-associated taxa, which may indirectly benefit cognitive health in CMD patients through improved metabolic signaling.

In our cohort, age, was the only host factor significantly influencing baseline gut microbiome structure, as shown by differences in beta diversity metrics. This aligns with previous reports highlighting age as a key determinant of microbiome composition.^66^^,^^67^ Intervention responses were heterogeneous, with no alpha diversity changes but beta diversity shifts in 68–73 y and 73–80 y.

In 60–64 y, increases in *Ruminococcus gnavus* group and *Parabacteroides* occurred alongside reductions in *Holdemanella*. The 65–68 group saw enrichments in *Oscillibacter* (estimate = 1.62; cholesterol-metabolizing) and *Lachnospiraceae UCG−003*. In 68–73 y, *Adlercreutzia* and *Slackia* (polyphenol degraders)^68^ increased, while *Barnesiella* decreased. The oldest group 73–80 y showed reductions in *Bifidobacterium*, *Lactobacillus*, and *Methanobrevibacter*, taxa often depleted in aged microbiomes but crucial for gut homeostasis. These age-dependent patterns reflect declining microbiome plasticity with advancing age, potentially limiting anthocyanin efficacy in older subgroups, yet the observed modulations of SCFA producers and anti-inflammatory taxa suggest benefits for neuroinflammation reduction and cognitive support via the gut-brain axis.

No direct effect of the intervention, nor modification by enterotype, BMI or age, was observed on QEM. However, several taxa exhibited correlations with cognitive outcomes. Increases in *Acetanaerobacterium* were linked to reduced QEM, whereas *Slackia*, a genus involved in polyphenol metabolism,^68^ was positively associated with improvements, consistent with anti-inflammatory actions relevant to cognition. Within the intervention group, *Phocea* and *Clostridium sensu stricto* 1 showed negative associations with QEM. Interestingly, although *Clostridium sensu stricto* 1 is generally considered protective and reduced in Alzheimer's patients,^69^ in our cohort, increases in this genus were associated with lower QEM, suggesting that stability rather than fluctuation may support cognitive function. Stratified analyses highlighted age-specific patterns: *Barnesiella* was positively correlated with QEM in the intervention group but negatively in the 73–80 age group, aligning with its association to cerebral small vessel disease burden, a risk factor for cognitive decline.^70^ *Moryella* showed a similar pattern (positive in intervention and negative in 65–68 y). *Moryella* has been implicated in Alzheimer's disease signatures via microbiota-gut-brain axis mediators.^71^ These associations underscore potential indirect links between anthocyanin-modulated taxa and cognition, possibly mediated via SCFA production, inflammation reduction, or neurogenesis. Nonetheless, mediation analyses indicated no significant role for these ∆CLR changes in mediating intervention effects on QEM, suggesting correlations rather than causation; larger studies are needed to clarify microbiome-cognition mediation in polyphenol interventions.

This study provides valuable insights, but limitations include the lack of dietary/antibiotic data, small subgroup sizes (*n* = 99), and no omics integration for functional pathways. Future work should use larger cohorts, metagenomics, and metabolomics to elucidate mechanisms, plus longitudinal designs to assess sustained effects. In conclusion, anthocyanins selectively modulate gut microbiota in older adults, with host factors driving variability and potential cognitive benefits via the gut-brain axis, supporting precision nutrition for mitigating aging-related risks.

## Supplementary Material

Supplementary material **Supplementary table S1:** Contaminant taxa identified using the prevalence-based method implemented in the decontam R package.**Supplementary table S2.** Description of study participants, including demographic characteristics, clinical conditions, and medication use.**Supplementary table S3.** Differentially abundant genera between enterotype groups identified using the Wilcoxon Signed-Rank Test.**Supplementary table S4.** Permutational multivariate analysis of variance (PERMANOVA) analysis of beta diversity metrics at baseline.**Supplementary table S5.** Statistical assessment of the overall effect of anthocyanin intervention on gut microbiome diversity at week 24.**Supplementary table S6.** Statistical assessment of the overall effect of anthocyanin intervention stratified by enterotype on gut microbiome diversity at week 24.**Supplementary table S7.** Statistical assessment of the overall effect of anthocyanin intervention stratified by body mass index (BMI) on gut microbiome diversity at week 24.**Supplementary table S8.** Statistical assessment of the overall effect of anthocyanin intervention stratified by age (quartiles) on gut microbiome diversity at week 24.**Supplementary table S9**. Analyses of cognitive performance (Quality Episodic Memory, QEM) at week 24.**Supplementary Table 10.** Differential associations between gut microbial taxa and Quality of Episodic Memory (QEM) in the intervention study, including overall and age-stratified effects.**Supplementary Figure S1.** Rarefaction curves of all samples in the study. The x-axis represents the library size, and the y-axis shows the amplicon sequence variant (ASV) richness at each library size. The red vertical dashed line indicates the optimal rarefaction depth, selected based on the plateau of ASV richness.**Supplementary Figure S2.** Determination of the optimal number of gut microbial community clusters using the Dirichlet Multinomial Mixture (DMM) model applied to genus-level rarefied count data. Model fit was evaluated using: (A) Laplace approximation, (B) Akaike Information Criterion (AIC), and (C) Bayesian Information Criterion (BIC). The optimal number of components was selected at the “elbow” point in the model-fit curve, where adding more components no longer substantially improves model fit but increased complexity, thus balancing fit and complexity. (D) Alluvial plot comparing clustering results between the two-cluster solution (selected by BIC) and the three-cluster solution (supported by AIC and Laplace). Each flow represents the number of samples shared between enterotypes across models. Stratum heights reflect the relative size of each enterotype, and flow widths indicate sample overlap. The plot illustrates areas of agreement and divergence between clustering solutions, highlighting how individual samples transition between enterotypes.

## Data Availability

Scripts used for data analysis are available at GitHub: https://github.com/yoh-s/ACID_Microbiome. Raw sequencing data are deposited in the National Center for Biotechnology Information (NCBI) Sequence Read Archive (SRA) under BioProject PRJNA1235737. Additional datasets and materials are available upon reasonable request from the corresponding author.
