# Supplementary material for: Gut enterotype- and body mass index (BMI)-dependent effects of anthocyanin supplementation on gut microbiota composition in individuals at risk for cognitive decline: a randomized placebo-controlled trial
Source: Gut Microbes. 2025 Oct 29;17(1):2570862. doi: 10.1080/19490976.2025.2570862 (PMC12578313; doi:10.1080/19490976.2025.2570862)
Supplement: Supplementary material — Supplementary table S1: Contaminant taxa identified using the prevalence-based method implemented in the decontam R package.Supplementary table S2. Description of study participants, including demographic characteristics, clinical conditions, and medication use.Supplementary table S3. Differentially abundant genera between enterotype groups identified using the Wilcoxon Signed-Rank Test.Supplementary table S4. Permutational multivariate analysis of variance (PERMANOVA) analysis of beta diversity metrics at baseline.Supplementary table S5. Statistical assessment of the overall effect of anthocyanin intervention on gut microbiome diversity at week 24.Supplementary table S6. Statistical assessment of the overall effect of anthocyanin intervention stratified by enterotype on gut microbiome diversity at week 24.Supplementary table S7. Statistical assessment of the overall effect of anthocyanin intervention stratified by body mass index (BMI) on gut microbiome diversity at week 24.Supplementary table S8. Statistical assessment of the overall effect of anthocyanin intervention stratified by age (quartiles) on gut microbiome diversity at week 24.Supplementary table S9. Analyses of cognitive performance (Quality Episodic Memory, QEM) at week 24.Supplementary Table 10. Differential associations between gut microbial taxa and Quality of Episodic Memory (QEM) in the intervention study, including overall and age-stratified effects.Supplementary Figure S1. Rarefaction curves of all samples in the study. The x-axis represents the library size, and the y-axis shows the amplicon sequence variant (ASV) richness at each library size. The red vertical dashed line indicates the optimal rarefaction depth, selected based on the plateau of ASV richness.Supplementary Figure S2. Determination of the optimal number of gut microbial community clusters using the Dirichlet Multinomial Mixture (DMM) model applied to genus-level rarefied count data. Model fit was evaluated using: (A) Laplace appro [file KGMI_A_2570862_SM3457.docx]

Supplementary materials to the research article titled “*Gut enterotype- and body mass index (BMI)-dependent effects of anthocyanin supplementation on gut microbiota composition in individuals at risk for cognitive decline: A randomized placebo-controlled trial*”

**Authors:** Yohannes Seyoum^a^, Chiara de Lucia^b,c^, Khadija Khalifab, Anne Katrine Bergland^b,d^, Dag Aarsland^b,c^, and Mark van der Giezen^a,e,f^

^a^Department of Chemistry, Bioscience, and Environmental Engineering, University of Stavanger (UiS), Stavanger, Norway

^b^Centre for Age-Related Medicine (SESAM), Stavanger University Hospital, Stavanger, Norway

^c^Centre for Healthy Brain Ageing, Department of Psychological Medicine, Institute of Psychiatry, Psychology and Neuroscience (IoPPN), King's College London, London, UK

^d^Department of Clinical Medicine, University of Bergen, Bergen, Norway

^e^Biosciences, University of Exeter, Exeter, UK

^f^Research Department, Stavanger University Hospital (SUS), Stavanger, Norway

**List of supplementary materials**

**Supplementary tables**

**Supplementary table S1:** Contaminant taxa identified using the prevalence-based method implemented in the decontam R package.

**Supplementary table S2.** Description of study participants, including demographic characteristics, clinical conditions, and medication use

**Supplementary table S3.** Differentially abundant genera between enterotype groups identified using the Wilcoxon Signed-Rank Test

**Supplementary table S4.** Permutational multivariate analysis of variance (PERMANOVA) analysis of beta diversity metrics at baseline

**Supplementary table S5**. Statistical assessment of the overall effect of anthocyanin intervention on gut microbiome diversity at week 24.

**Supplementary table S6**. Statistical assessment of the overall effect of anthocyanin intervention stratified by enterotype on gut microbiome diversity at week 24.

**Supplementary table S7.** Statistical assessment of the overall effect of anthocyanin intervention stratified by body mass index (BMI) on gut microbiome diversity at week 24.

**Supplementary table S8.** Statistical assessment of the overall effect of anthocyanin intervention stratified by age (quartiles) on gut microbiome diversity at week 24.

**Supplementary table S9.** Analyses of cognitive performance (Quality Episodic Memory, QEM) at week 24

**Supplementary Table 10.** Differential associations between gut microbial taxa and Quality of Episodic Memory (QEM) in the intervention study, including overall and age-stratified effects.

**Supplementary figures**

**Supplementary Figure S1.** Rarefaction curves of all samples in the study. The x-axis represents the library size, and the y-axis shows the amplicon sequence variant (ASV) richness at each library size. The red vertical dashed line indicates the optimal rarefaction depth, selected based on the plateau of ASV richness.

**Supplementary Figure S2.** Supplementary Figure S2. Determination of the optimal number of gut microbial community clusters using the Dirichlet Multinomial Mixture (DMM) model applied to genus-level rarefied count data. Model fit was evaluated using: (A) Laplace approximation, (B) Akaike Information Criterion (AIC), and (C) Bayesian Information Criterion (BIC). The optimal number of components was selected at the “elbow” point in the model-fit curve, where adding more components no longer substantially improves model fit but increased complexity, thus balancing fit and complexity. (D) Alluvial plot comparing clustering results between the two-cluster solution (selected by BIC) and the three-cluster solution (supported by AIC and Laplace). Each flow represents the number of samples shared between enterotypes across models. Stratum heights reflect the relative size of each enterotype, and flow widths indicate sample overlap. The plot illustrates areas of agreement and divergence between clustering solutions, highlighting how individual samples transition between enterotypes.

**Supplementary Figure S3**. Stacked bar plot showing the mean relative abundance (%) of species within the Bacteroides genus across enterotype one and enterotype two. Each bar represents the average composition of Bacteroides species within the respective enterotype, highlighting differences in species-level distribution between the two microbial community types.

**Supplementary tables**

**Supplementary table S1:** Contaminant taxa identified using the prevalence-based method implemented in the decontam R package.

| **Family** | **Genus** | **Species** |
| --- | --- | --- |
| Clostridiaceae | *Clostridium_sensu_stricto_1* | Unclassified |
| Beijerinckiaceae | *Bosea* | Unclassified |
| Lachnospiraceae | *uncultured* | Unclassified |
| Ruminococcaceae | *Faecalibacterium* | Unclassified |
| Erysipelatoclostridiaceae | *Asteroleplasma* | uncultured_bacterium |
| Lachnospiraceae | *Coprococcus* | Unclassified |
| Bacteroidaceae | *Bacteroides* | *Bacteroides_fragilis* |
| Clostridia_UCG-014 | *Clostridia_UCG-014* | Unclassified |
| Lachnospiraceae | *Lachnospiraceae_ND3007_group* | uncultured_bacterium |
| Propionibacteriaceae | *Cutibacterium* | Unclassified |
| Xanthomonadaceae | *Lysobacter* | Unclassified |
| Alcaligenaceae | *Alcaligenes* | Unclassified |
| Clostridiaceae | *Clostridium_sensu_stricto_1* | Unclassified |
| Bacillaceae | *Bacillus* | Unclassified |
| Lachnospiraceae | *Roseburia* | Unclassified |
| Bacteroidaceae | *Bacteroides* | *Bacteroides_vulgatus* |
| Lachnospiraceae | Unclassified | Unclassified |
| Enterococcaceae | *Enterococcus* | Unclassified |
| Streptococcaceae | *Streptococcus* | Unclassified |
| Monoglobaceae | *Monoglobus* | Unclassified |
| Rhizobiaceae | *Allorhizobium-Neorhizobium-Pararhizobium-Rhizobium* | Unclassified |
| Bifidobacteriaceae | *Bifidobacterium* | *Bifidobacterium_breve* |
| Prevotellaceae | *Prevotella_9* | Unclassified |
| Ruminococcaceae | *Ruminococcus* | *Ruminococcus_bicirculans* |
| Enterobacteriaceae | Unclassified | Unclassified |
| Gastranaerophilales | *Gastranaerophilales* | gut_metagenome |
| Ruminococcaceae | Unclassified | Unclassified |
| Lachnospiraceae | *Agathobacter* | Unclassified |
| Rhizobiales_Incertae_Sedis | *Phreatobacter* | Unclassified |
| UCG-010 | *UCG-010* | uncultured_bacterium |
| Xanthomonadaceae | *Stenotrophomonas* | Unclassified |
| Staphylococcaceae | *Staphylococcus* | Unclassified |
| Bifidobacteriaceae | *Gardnerella* | *Gardnerella_vaginalis* |
| Barnesiellaceae | *Barnesiella* | Unclassified |

**Supplementary table S2.** Description of study participants, including demographic characteristics, clinical conditions, and medication use

| **Variable** | **Baseline** | | | | **Week 12** | | | | **Week 24** | | | |
| --- | --- | --- | --- | --- | --- | --- | --- | --- | --- | --- | --- | --- |
|  | Treatment A (N=45) | ***N*** | Treatment B (N=54) | ***N*** | Treatment A (N=44) | ***N*** | Treatment B (N=53) | ***N*** | Treatment A (N=43) | ***N*** | Treatment B (N=51) | ***N*** |
| APOE ε3/4 heterozygote/ε4/4 homozygote | 31.1 | 45 | 44.4 | 54 | 31.8 | 44 | 43.4 | 53 | 34.9 | 43 | 43.1 | 51 |
| Cardiometabolic disease (CMD) | 75.6 | 45 | 74.1 | 54 | 79.5 | 44 | 71.7 | 53 | 74.4 | 43 | 74.5 | 51 |
| Residence |  | 45 |  | 54 |  | 44 |  | 53 |  | 43 |  | 51 |
| Bergen | 37.8 |  | 38.9 |  | 40.9 |  | 41.5 |  | 39.5 |  | 39.2 |  |
| Stavanger | 62.2 |  | 57.4 |  | 59.1 |  | 58.5 |  | 60.5 |  | 60.8 |  |
| Gender (Women) | 48.9 | 45 | 53.7 | 54 | 45.5 |  | 50.9 |  | 46.5 |  | 51 |  |
| Age – median (IQR) | 68(5) | 45 | 70(9) | 54 | 68(5) | 44 | 69(9) | 53 | 68(5) | 43 | 68(5) | 51 |
| Age (quartile classification) |  |  |  |  |  |  |  |  |  |  |  |  |
| 60 – 64 years | 28.9 |  | 22.2 |  | 29.5 |  | 22.6 |  | 30.2 |  | 23.5 |  |
| 65 – 68 years | 42.2 |  | 16.7 |  | 38.6 |  | 17 |  | 41.9 |  | 17.6 |  |
| 68 – 73 years | 11.1 |  | 31.5 |  | 15.9 |  | 32.1 |  | 11.6 |  | 29.4 |  |
| 73 – 80 years | 17.8 |  | 29.6 |  | 15.9 |  | 28.3 |  | 16.3 |  | 29.4 |  |
| Marital status |  | 45 |  | 54 |  | 44 |  | 53 |  | 43 |  | 51 |
| Married | 66.7 |  | 74.1 |  | 68.2 |  | 73.6 |  | 67.4 |  | 78.4 |  |
| Single | 17.8 |  | 11.1 |  | 15.9 |  | 11.3 |  | 16.3 |  | 9.8 |  |
| Divorced | 15.6 |  | 14.8 |  | 15.9 |  | 15.1 |  | 16.3 |  | 11.8 |  |
| Familial dementia | 68.9 | 45 | 63 | 54 | 72.7 | 44 | 62.3 | 53 | 67.4 | 43 | 64.7 | 51 |
| Smoker | 8.9 | 45 | 7.4 | 54 | 9.1 | 44 | 7.5 | 53 | 11.6 | 43 | 7.8 | 51 |
| Drinking alcohol | 91.1 | 45 | 85.2 | 54 | 88.6 | 44 | 86.8 | 53 | 88.4 | 43 | 84.3 | 51 |
| Coronary heart disease | 13.3 | 45 | 11.1 | 54 | 13.6 | 44 | 11.3 | 53 | 16.3 | 43 | 11.8 | 51 |
| Hypertension treatment | 68.9 | 45 | 64.8 | 54 | 68.2 | 44 | 62.3 | 53 | 65.1 | 43 | 62.7 | 51 |
| Hypercholesterolemia drug | 53.3 | 45 | 63 | 54 | 52.3 | 44 | 62.3 | 53 | 48.8 | 43 | 58.8 | 51 |
| Metabolic disease | 11.1 | 45 | 14.8 | 54 | 6.8 | 44 | 13.2 | 53 | 11.6 | 43 | 11.8 | 51 |
| Diabetes mellitus | 22.2 | 45 | 18.5 | 54 | 25 | 44 | 17 | 53 | 20.9 | 43 | 19.6 | 51 |
| BMI, kg/m^2^ - median (IQR) | 28.2(4.9) | 45 | 28.8(5.9) | 54 | 27.6 (3.8) | 44 | 29.9(6.6) | 46 | 27.8(4.8) | 40 | 27.8(4.8) | 48 |
| BMI classification |  |  |  |  |  |  |  |  |  |  |  |  |
| Overweight | 51.1 |  | 44.4 |  | 22 | 41 | 45.7 |  | 52.5 |  | 39.6 |  |
| Obesity | 22.2 |  | 37 |  | 58.5 | 41 | 34.8 |  | 27.5 |  | 39.6 |  |
| Diabetes medication (Yes) | 24.4 | 45 | 14 | 50 | 25.6 | 40 | 14.3 | 49 | 20.9 | 43 | 14.9 | 47 |
| Antithrombotic agents (Yes) | 33.3 | 45 | 38 | 50 | 34.9 | 40 | 36.7 | 49 | 34.9 | 43 | 38.3 | 47 |
| Calcium channel blockers (Yes) | 40 | 45 | 40 | 50 | 39.5 | 40 | 38.8 | 49 | 39.5 | 43 | 36.2 | 47 |
| Anti-inflammatory antirheumatic drugs (Yes) | 2.2 | 45 | 4 | 50 | 2.3 | 40 | 4.1 | 49 | 2.3 | 43 | 4.3 | 47 |
| Thyroid therapy (Yes) | 11.1 | 45 | 10 | 50 | 7 | 40 | 8.2 | 49 | 11.6 | 43 | 6.4 | 47 |
| Proton pump inhibitors (Yes) | 17.8 | 45 | 10 | 50 | 18.6 | 40 | 6.1 | 49 | 16.3 | 43 | 10.6 | 47 |

**Supplementary table S3.** Differentially abundant genera between enterotype groups identified using the Wilcoxon Signed-Rank Test

| **Genus** | **Enterotype one (N = 65)** | **Enterotype two (N = 31)** | **statistic** | **BH P value** |
| --- | --- | --- | --- | --- |
| *UCG-010* | 0.58 | 0.14 | 1673 | 0.00004 |
| *Clostridia_vadinBB60_group* | 0.69 | 0.15 | 1642.5 | 0.0001 |
| *UCG-005* | 1.38 | 0.56 | 1616 | 0.0001 |
| *UCG-009* | 0.02 | 0.004 | 1571 | 0.0002 |
| *Izemoplasmatales* | 0.16 | 0.03 | 1523 | 0.0007 |
| *Christensenellaceae_R-7_group* | 3.32 | 1.58 | 1545 | 0.0007 |
| *uncultured_Ruminococcaceae* | 0.46 | 0.21 | 1545 | 0.0007 |
| *Lachnospiraceae_UCG-008* | 0.12 | 0.05 | 1530 | 0.0010 |
| *Alistipes* | 3.39 | 1.41 | 1506 | 0.002 |
| *Lachnospiraceae_FCS020_group* | 0.12 | 0.06 | 1492 | 0.003 |
| *[Eubacterium]_ventriosum_group* | 0.21 | 0.10 | 1478 | 0.004 |
| *uncultured_Rhodospirillales* | 0.96 | 0.29 | 1449.5 | 0.004 |
| *Eggerthella* | 0.002 | 0.02 | 653.5 | 0.004 |
| *Moryella* | 0.04 | 0.02 | 1460 | 0.004 |
| *UCG-002* | 3.87 | 2.34 | 1460 | 0.005 |
| *Faecalitalea* | 8.08E-05 | 0.03 | 793 | 0.006 |
| *UCG-003* | 0.37 | 0.16 | 1446 | 0.006 |
| *Odoribacter* | 0.20 | 0.12 | 1446.5 | 0.006 |
| Butyricicoccus | 0.29 | 0.15 | 1443 | 0.007 |
| Clostridia_UCG-014 | 3.05 | 2.47 | 1438 | 0.007 |
| Coprococcus | 3.29 | 1.95 | 1426 | 0.010 |
| uc_Oscillospiraceae | 0.17 | 0.08 | 1422 | 0.010 |
| Marvinbryantia | 0.13 | 0.06 | 1412 | 0.01 |
| NK4A214_group | 1.57 | 0.98 | 1405 | 0.02 |
| Lachnospiraceae_UCG-004 | 0.17 | 0.07 | 1393 | 0.02 |
| Methanobrevibacter | 0.90 | 0.51 | 1355 | 0.02 |
| RF39 | 0.18 | 0.04 | 1363.5 | 0.02 |
| Lachnospiraceae_ND3007_group | 0.57 | 0.24 | 1382 | 0.02 |
| [Ruminococcus]_gnavus_group | 0.04 | 1.17 | 703 | 0.03 |
| Tuzzerella | 0.003 | 0 | 1240 | 0.03 |
| vadinBE97 | 0.04 | 0.01 | 1262 | 0.03 |
| Sellimonas | 0.0004 | 0.005 | 860 | 0.04 |
| UCG-007 | 0.01 | 0.003 | 1324.5 | 0.04 |
| DTU014 | 0.006 | 0.002 | 1298 | 0.04 |
| Sutterella | 0.50 | 0.33 | 1343.5 | 0.04 |
| GCA-900066575 | 0.07 | 0.05 | 1354 | 0.04 |
| CAG-56 | 0.27 | 0.18 | 1347 | 0.04 |
| [Ruminococcus]_gauvreauii_group | 0.11 | 0.10 | 1346 | 0.04 |
| Fournierella | 0.08 | 0.03 | 1326.5 | 0.04 |
| Ruminiclostridium | 0.02 | 0.004 | 1255.5 | 0.04 |
| Faecalibacterium | 9.80 | 7.82 | 1342 | 0.04 |

Differences between the two enterotype groups were assessed using the Wilcoxon rank-sum test. P-values were adjusted for multiple comparisons using the Benjamini-Hochberg (BH) procedure to control the false discovery rate.

**Supplementary table S4.** Permutational multivariate analysis of variance (PERMANOVA) analysis of beta diversity metrics at baseline

| **Bray-Curtis dissimilarity (genus-level relative abundance)** | | | | | | |
| --- | --- | --- | --- | --- | --- | --- |
| **Variables** | **Degree of freedom** | **Sum of squares** | **R^2^** | **Effect size (%)** | **F** | **P value** |
| Age (quartile) | 3 | 0.74 | 0.044 | 4.40 | 1.41 | 0.04 |
| Screening diagnosis | 1 | 0.1203 | 0.007 | 0.72 | 0.68 | 0.85 |
| Residence | 1 | 0.1952 | 0.012 | 1.16 | 1.11 | 0.35 |
| Gender | 1 | 0.15 | 0.009 | 0.89 | 0.85 | 0.64 |
| BMI category | 2 | 0.3559 | 0.021 | 2.12 | 1.01 | 0.43 |
| Coronary heart disease | 1 | 0.1596 | 0.010 | 0.95 | 0.90 | 0.53 |
| Hypercholesterolemia drug | 1 | 0.1312 | 0.008 | 0.78 | 0.74 | 0.77 |
| Thyroid therapy | 1 | 0.1608 | 0.010 | 0.99 | 0.90 | 0.54 |
| Family dementia | 1 | 0.1966 | 0.012 | 1.17 | 1.11 | 0.29 |
| Hypertension treatment | 1 | 0.2187 | 0.013 | 1.30 | 1.24 | 0.20 |
| Diabetes mellitus all | 1 | 0.2452 | 0.015 | 1.46 | 1.39 | 0.12 |
| Antithrombotic agents | 1 | 0.088 | 0.005 | 0.54 | 0.49 | 0.97 |
| Drugs (acid related disorders) | 1 | 0.2484 | 0.015 | 1.53 | 0.13 | 0.13 |
| **Bray-Curtis dissimilarity (genus-level relative abundance) - Pairwise test using adonis2** | | | | | | |
| Age (65-68) vs Age (68-73) | 1 | 0.3874 | 0.04 | 4.26 | 2.091 | 0.01 |
| Age (60-64) vs Age (68-73) | 1 | 0.3169 | 0.32 | 31.69 | 1.711 | 0.05 |
| Age (65-68) vs Age (73-80) | 1 | 0.1935 | 0.023 | 2.34 | 1.172 | 0.25 |
| Age (65-68) vs Age (60-64) | 1 | 0.1383 | 0.017 | 1.71 | 0.832 | 0.68 |
| Age (73-80) vs Age (60-64) | 1 | 0.2394 | 0.03 | 2.88 | 1.248 | 0.19 |
| Age (60-64) vs Age (68-73) | 1 | 0.2394 | 0.028 | 2.88 | 1.305 | 0.18 |
| Enterotype one vs enterotype two | 1 | 1.69 | 0.155 | 15.6 | 14.18 | 0.001 |
| Enterotype one vs enterotype three | 1 | 1.02 | 0.102 | 10.2 | 6.7 | 0.001 |
| Enterotype two vs enterotype three | 1 | 0.93 | 0.082 | 8.2 | 4.998 | 0.001 |
| **Bray-Curtis dissimilarity (ASV level)** | | | | | | |
| Screening diagnosis | 1 | 0.2654 | 0.009 | 0.85 | 0.81 | 0.92 |
| Residence | 1 | 0.2959 | 0.009 | 0.95 | 0.90 | 0.74 |
| Age (quartile) | 3 | 1.1027 | 0.035 | 3.54 | 1.12 | 0.08 |
| Gender | 1 | 0.2711 | 0.008 | 0.82 | 0.82 | 0.89 |
| BMI category | 2 | 0.7248 | 0.232 | 23.23 | 1.11 | 0.15 |
| Coronary heart disease | 1 | 0.2815 | 0.009 | 0.90 | 0.86 | 0.85 |
| Hypercholesterolemia drug | 1 | 0.278 | 0.009 | 0.89 | 0.85 | 0.87 |
| Thyroid therapy | 1 | 0.3158 | 0.011 | 1.05 | 0.96 | 0.57 |
| Family dementia | 1 | 0.3628 | 0.012 | 1.16 | 1.11 | 0.22 |
| Hypertension treatment | 1 | 0.3609 | 0.012 | 1.16 | 1.10 | 0.23 |
| Diabetes mellitus all | 1 | 0.421 | 0.013 | 1.35 | 1.29 | 0.05 |
| Antithrombotic agents | 1 | 0.2353 | 0.008 | 0.79 | 0.71 | 0.99 |
| Drugs (acid related disorders) | 1 | 0.3309 | 0.011 | 1.10 | 1.01 | 0.42 |
| **Jaccard distance (ASV level)** | | | | | | |
| Screening Diagnosis | 1 | 0.414 | 0.010 | 0.98 | 0.93 | 0.94 |
| Residence | 1 | 0.43 | 0.010 | 1.02 | 0.97 | 0.72 |
| Age (quartile) | 3 | 1.377 | 0.033 | 3.26 | 1.03 | 0.11 |
| Gender | 1 | 0.42 | 0.010 | 1.00 | 0.95 | 0.88 |
| BMI category | 2 | 0.912 | 0.022 | 2.16 | 1.03 | 0.19 |
| Coronary heart disease | 1 | 0.426 | 0.010 | 1.01 | 0.96 | 0.82 |
| Hypercholesterolemia drug | 1 | 0.421 | 0.010 | 1.00 | 0.95 | 0.89 |
| Thyroid therapy | 1 | 0.436 | 0.011 | 1.08 | 0.98 | 0.62 |
| Family dementia | 1 | 0.461 | 0.011 | 1.09 | 1.04 | 0.18 |
| Hypertension treatment | 1 | 0.46 | 0.011 | 1.09 | 1.04 | 0.21 |
| Diabetes mellitus all | 1 | 0.484 | 0.011 | 1.15 | 1.09 | 0.05 |
| Antithrombotic agents | 1 | 0.405 | 0.010 | 1.00 | 0.91 | 0.99 |
| Drugs (acid related disorders) | 1 | 0.422 | 0.011 | 1.09 | 1.00 | 0.48 |
| **Weighted UniFrac distance (ASV level)** | | | | | | |
| Age (quartile) | 3 | 0.08183 | 0.067 |  | 2.190 | 0.005 |
| Screening Diagnosis | 1 | 0.00608 | 0.005 |  | 0.468 | 0.92 |
| Residence | 1 | 0.01643 | 0.013 |  | 1.275 | 0.23 |
| Gender | 1 | 0.00896 | 0.007 |  | 0.691 | 0.72 |
| BMI category | 2 | 0.03581 | 0.029 |  | 1.397 | 0.13 |
| Coronary heart disease | 1 | 0.0101 | 0.008 |  | 0.779 | 0.61 |
| Hypercholesterolemia drug | 1 | 0.00592 | 0.005 |  | 0.455 | 0.94 |
| Thyroid therapy | 1 | 0.01162 | 0.010 |  | 0.891 | 0.47 |
| Family dementia | 1 | 0.02288 | 0.019 |  | 1.785 | 0.08 |
| Hypertension treatment | 1 | 0.01483 | 0.012 |  | 1.150 | 0.29 |
| Diabetes mellitus all | 1 | 0.01591 | 0.013 |  | 1.234 | 0.25 |
| Antithrombotic agents | 1 | 0.00563 | 0.005 |  | 0.430 | 0.96 |
| Drugs (acid related disorders) | 1 | 0.01134 | 0.010 |  | 0.868 | 0.53 |
| **Weighted UniFrac distance (ASV level)** | | | | | | |
| Age (quartile) | 3 | 0.08183 | 0.067 |  | 2.190 | 0.005 |
| Screening Diagnosis | 1 | 0.00608 | 0.005 |  | 0.468 | 0.92 |
| Residence | 1 | 0.01643 | 0.013 |  | 1.275 | 0.23 |
| Gender | 1 | 0.00896 | 0.007 |  | 0.691 | 0.72 |
| BMI category | 2 | 0.03581 | 0.029 |  | 1.397 | 0.13 |
| Coronary heart disease | 1 | 0.0101 | 0.008 |  | 0.779 | 0.61 |
| Hypercholesterolemia drug | 1 | 0.00592 | 0.005 |  | 0.455 | 0.94 |
| Thyroid therapy | 1 | 0.01162 | 0.010 |  | 0.891 | 0.47 |
| Family dementia | 1 | 0.02288 | 0.019 |  | 1.785 | 0.08 |
| Hypertension treatment | 1 | 0.01483 | 0.012 |  | 1.150 | 0.29 |
| Diabetes mellitus all | 1 | 0.01591 | 0.013 |  | 1.234 | 0.25 |
| Antithrombotic agents | 1 | 0.00563 | 0.005 |  | 0.430 | 0.96 |
| Drugs (acid related disorders) | 1 | 0.01134 | 0.010 |  | 0.868 | 0.53 |
| **Weighted UniFrac distance (ASV level) - Pairwise test using adonis2** | | | | | | |
| Age (65-68) vs Age (73-80) | 1 | 0.02739 | 0.04 | 4.4 | 2.242 | 0.03 |
| Age (65-68) vs Age (60-64) | 1 | 0.01356 | 0.02 | 2.3 | 1.118 | 0.28 |
| Age (65-68) vs Age (68-73) | 1 | 0.05102 | 0.08 | 7.6 | 3.854 | 0.004 |
| Age (73-80) vs Age (60-64) | 1 | 0.01545 | 0.03 | 2.9 | 1.328 | 0.20 |
| Age (60-64) vs Age (68-73) | 1 | 0.02227 | 0.04 | 3.8 | 1.739 | 0.08 |
| Age (60-64) vs Age (68-73) | 1 | 0.03399 | 0.06 | 5.8 | 2.671 | 0.008 |
| **Unweighted UniFrac distance (ASV level)** | | | | | | |
| Screening Diagnosis | 1 | 0.3138 | 0.010 | 1.01 | 0.96 | 0.50 |
| Residence | 1 | 0.3168 | 0.010 | 1.02 | 0.97 | 0.50 |
| Age (quartile) | 3 | 1.107 | 0.036 | 3.56 | 1.13 | 0.11 |
| Gender | 1 | 0.3248 | 0.010 | 1.05 | 0.99 | 0.44 |
| BMI category | 2 | 0.7076 | 0.023 | 2.28 | 1.08 | 0.22 |
| Coronary heart disease | 1 | 0.2927 | 0.009 | 0.94 | 0.89 | 0.70 |
| Hypercholesterolemia drug | 1 | 0.2967 | 0.010 | 0.96 | 0.91 | 0.72 |
| Thyroid therapy | 1 | 0.4032 | 0.014 | 1.36 | 1.24 | 0.08 |
| Family dementia | 1 | 0.3112 | 0.010 | 1.00 | 0.95 | 0.55 |
| Hypertension treatment | 1 | 0.357 | 0.011 | 1.15 | 1.09 | 0.20 |
| Diabetes mellitus all | 1 | 0.4014 | 0.013 | 1.29 | 1.23 | 0.08 |
| Antithrombotic agents | 1 | 0.2876 | 0.010 | 0.97 | 0.88 | 0.78 |
| Drugs (acid related disorders) | 1 | 0.3539 | 0.012 | 1.20 | 1.09 | 0.22 |

**Supplementary table S5.** Statistical assessment of the overall effect of anthocyanin intervention on gut microbiome diversity at week 24.

| **Analysis of covariance (ANCOVA) - alpha diversity metrics** | | | | | | |
| --- | --- | --- | --- | --- | --- | --- |
|  | **Estimate** | **Standard error** | **Statistic** | **P adjusted** | | |
| Observed richness | 4.94 | 10.84 | 0.46 | 0.95 | | |
| Shannon diversity | 0.007 | 0.11 | 0.064 | 0.95 | | |
| Faith’s phylogenetic diversity | 0.12 | 0.53 | 0.23 | 0.95 | | |
| Pielou’s evenness | 0.00091 | 0.016 | 0.058 | 0.95 | | |
| **Permutational analysis of variance (PERMANOVA) - Beta diversity** | | | | | | |
|  | **Degree of freedom** | **Sum of squares** | **R^2^** | **Effect size (%)** | **F** | **P value** |
| Bray-Curtis (genus level) | 3 | 0.43 | 0.013 | 1.3 | 0.78 | 0.061 |
| Bray-Curtis (ASV level) | 3 | 0.74 | 0.013 | 1.3 | 0.74 | 0.01 |
| Jaccard distance | 3 | 0.76 | 0.014 | 1.4 | 0.79 | 0.036 |
| Weighted UniFrac | 3 | 0.0013 | 0.012 | 1.2 | 0.71 | 0.21 |
| Unweighted UniFrac | 3 | 0.73 | 0.013 | 1.3 | 0.78 | 0.002 |

Alpha diversity was evaluated using analysis of covariance (ANCOVA) for observed richness, Shannon diversity, Faith’s phylogenetic diversity, and Pielou’s evenness, adjusting for baseline values. Beta diversity was assessed with permutational analysis of variance (PERMANOVA) at both genus and ASV levels using Bray-Curtis, Jaccard, Weighted UniFrac, and Unweighted UniFrac distances. The table reports estimates, standard errors, test statistics, adjusted P-values for ANCOVA, and degrees of freedom, sum of squares, R², effect size (%), F statistic, and P values for PERMANOVA.

**Supplementary table S6.** Statistical assessment of the overall effect of anthocyanin intervention stratified by enterotype on gut microbiome diversity at week 24.

| **Analysis of covariance (ANCOVA) - alpha diversity metrics** | | | | | | |
| --- | --- | --- | --- | --- | --- | --- |
|  | **Estimate** | **Standard error** | **Statistic** | **P adjusted** | | |
| **Observed richness** |  |  |  |  | | |
| Enterotype one | 2.45 | 12.62 | 0.19 | 0.85 | | |
| Enterotype two | -7.85 | 19.41 | -0.40 | 0.85 | | |
| **Shannon diversity** |  |  |  |  | | |
| Enterotype one | 0.13 | 0.10 | 1.30 | 0.34 | | |
| Enterotype two | -0.15 | 0.16 | -0.96 | 0.34 | | |
| **Faith’s phylogenetic diversity** |  |  |  |  | | |
| Enterotype one | 0.29 | 0.60 | 0.48 | 0.83 | | |
| Enterotype two | -0.19 | 0.92 | -0.21 | 0.83 | | |
| **Pielou’s evenness** |  |  |  |  | | |
| Enterotype one | 0.021 | 0.01 | 1.48 | 0.28 | | |
| Enterotype two | -0.023 | 0.02 | -1.05 | 0.30 | | |
| **Permutational analysis of variance (PERMANOVA) - Beta diversity** | | | | | | |
|  | **Degree of freedom** | **Sum of squares** | **R^2^** | **Effect size (%)** | **F value** | **P value** |
| **Bray-Curtis (genus level)** |  |  |  |  |  |  |
| Enterotype one | 3 | 0.34 | 0.018 |  | 0.72 | 0.26 |
| Enterotype two | 3 | 0.71 | 0.059 |  | 1.06 | 0.006 |
| **Bray-Curtis (ASV level)** |  |  |  |  |  |  |
| Enterotype one | 3 | 0.65 | 0.018 |  | 0.70 | 0.071 |
| Enterotype two | 3 | 0.97 | 0.050 |  | 0.88 | 0.021 |
| **Jaccard distance** |  |  |  |  |  |  |
| Enterotype one | 3 | 0.68 | 0.019 |  | 0.75 | 0.058 |
| Enterotype two | 3 | 0.82 | 0.045 |  | 0.79 | 0.066 |
| **Weighted UniFrac** |  |  |  |  |  |  |
| Enterotype one | 3 | 0.002 | 0.024 |  | 0.97 | 0.21 |
| Enterotype two | 3 | 0.001 | 0.067 |  | 1.21 | 0.014 |
| **Unweighted UniFrac** |  |  |  |  |  |  |
| Enterotype one | 3 | 0.669 | 0.002 |  | 0.78 | 0.006 |
| Enterotype two | 3 | 0.849 | 0.045 |  | 0.79 | 0.003 |

Alpha diversity was evaluated using analysis of covariance (ANCOVA) for observed richness, Shannon diversity, Faith’s phylogenetic diversity, and Pielou’s evenness, adjusting for baseline values. Beta diversity was assessed with permutational analysis of variance (PERMANOVA) at both genus and ASV levels using Bray-Curtis, Jaccard, Weighted UniFrac, and Unweighted UniFrac distances. The table reports estimates, standard errors, test statistics, adjusted P-values for ANCOVA, and degrees of freedom, sum of squares, R², effect size (%), F statistic, and P values for PERMANOVA.

**Supplementary table S7.** Statistical assessment of the overall effect of anthocyanin intervention stratified by body mass index (BMI) on gut microbiome diversity at week 24.

| **Analysis of covariance (ANCOVA) - alpha diversity metrics** | | | | | | |
| --- | --- | --- | --- | --- | --- | --- |
|  | **Estimate** | **Standard error** | **Statistic** | **P adjusted** | | |
| **Observed richness** |  |  |  |  | | |
| Healthy weight | -7.40 | 22.65 | -0.33 | 0.74 | | |
| Overweight | -13.08 | 15.21 | -0.86 | 0.59 | | |
| Obesity |  |  |  |  | | |
| **Shannon diversity** |  |  |  |  | | |
| Healthy weight | -0.19 | 0.18 | -1.04 | 0.46 | | |
| Overweight | 0.15 | 0.12 | 1.17 | 0.46 | | |
| Obesity | 0.10 | 0.16 | 0.59 | 0.56 | | |
| **Faith’s phylogenetic diversity** |  |  |  |  | | |
| Healthy weight | -0.29 | 1.08 | -0.26 | 0.95 | | |
| Overweight | -0.04 | 0.73 | -0.06 | 0.95 | | |
| Obesity | 0.83 | 0.96 | 0.87 | 0.95 | | |
| **Pielou’s evenness** |  |  |  |  | | |
| Healthy weight | -0.03 | 0.025 | -1.25 | 0.32 | | |
| Overweight | 0.03 | 0.017 | 1.89 | 0.19 | | |
| Obesity | 0.001 | 0.022 | 0.053 | 0.96 | | |
| **Permutational analysis of variance (PERMANOVA) - Beta diversity** | | | | | | |
|  | **Degree of freedom** | **Sum of squares** | **R^2^** | **Effect size (%)** | **F value** | **P value** |
| **Bray-Curtis (genus level)** |  |  |  |  |  |  |
| Healthy weight | 3 | 0.39 | 0.06 | 6.0 | 0.76 | 0.84 |
| Overweight | 3 | 0.40 | 0.029 | 2.9 | 0.77 | 0.97 |
| Obesity | 3 | 0.55 | 0.051 | 5.1 | 0.90 | 0.23 |
| **Bray-Curtis (ASV level)** |  |  |  |  |  |  |
| Healthy weight | 3 | 0.78 | 0.064 | 6.4 | 0.78 | 0.70 |
| Overweight | 3 | 0.80 | 0.031 | 3.1 | 0.83 | 0.12 |
| Obesity | 3 | 0.79 | 0.044 | 4.4 | 0.77 | 0.66 |
| **Jaccard distance** |  |  |  |  |  |  |
| Healthy weight | 3 | 0.81 | 0.068 | 6.8 | 0.83 | 0.77 |
| Overweight | 3 | 0.71 | 0.029 | 2.9 | 0.78 | 0.29 |
| Obesity | 3 | 0.78 | 0.045 | 4.5 | 0.78 | 0.19 |
| **Weighted UniFrac** |  |  |  |  |  |  |
| Healthy weight | 3 | 0.0007 | 0.044 | 4.4 | 0.52 | 0.12 |
| Overweight | 3 | 0.0013 | 0.022 | 2.2 | 0.58 | 0.71 |
| Obesity | 3 | 0.0015 | 0.05 | 5.0 | 0.88 | 0.67 |
| **Unweighted UniFrac** |  |  |  |  |  |  |
| Healthy weight | 3 | 0.76 | 0.065 | 6.5 | 0.79 | 0.41 |
| Overweight | 3 | 0.67 | 0.029 | 2.9 | 0.75 | 0.64 |
| Obesity | 3 | 0.74 | 0.043 | 4.3 | 0.75 | 0.02 |

Alpha diversity was evaluated using analysis of covariance (ANCOVA) for observed richness, Shannon diversity, Faith’s phylogenetic diversity, and Pielou’s evenness, adjusting for baseline values. Beta diversity was assessed with permutational analysis of variance (PERMANOVA) at both genus and ASV levels using Bray-Curtis, Jaccard, Weighted UniFrac, and Unweighted UniFrac distances. The table reports estimates, standard errors, test statistics, adjusted P-values for ANCOVA, and degrees of freedom, sum of squares, R², effect size (%), F statistic, and P values for PERMANOVA.

**Supplementary table S8.** Statistical assessment of the overall effect of anthocyanin intervention stratified by age (quartiles) on gut microbiome diversity at week 24.

| **Analysis of covariance (ANCOVA) - alpha diversity metrics** | | | | | | |
| --- | --- | --- | --- | --- | --- | --- |
|  | **Estimate** | **Standard error** | **Statistic** | **P adjusted** | | |
| **Observed richness** |  |  |  |  | | |
| 60 - 64 years | 19.41 | 21.42 | 0.91 | 0.40 | | |
| 65- 68 years | -16.62 | 19.52 | -0.85 | 0.40 | | |
| 68 - 73 years | -26.24 | 26.64 | -0.99 | 0.40 | | |
| 73 - 80 years | 29.49 | 21.73 | 1.36 | 0.40 | | |
| **Shannon diversity** |  |  |  |  | | |
| 60 - 64 years | 0.21 | 0.18 | 1.20 | 0.58 | | |
| 65- 68 years | -0.13 | 0.16 | -0.79 | 0.58 | | |
| 68 - 73 years | 0.05 | 0.22 | 0.22 | 0.83 | | |
| 73 - 80 years | 0.18 | 0.18 | 0.99 | 0.58 | | |
| **Faith’s phylogenetic diversity** |  |  |  |  | | |
| 60 - 64 years | 1.09 | 1.02 | 1.07 | 0.38 | | |
| 65- 68 years | -0.66 | 0.93 | -0.71 | 0.48 | | |
| 68 - 73 years | -1.93 | 1.25 | -1.54 | 0.25 | | |
| 73 - 80 years | 1.85 | 1.03 | 1.79 | 0.25 | | |
| **Pielou’s evenness** |  |  |  |  | | |
| 60 - 64 years | 0.03 | 0.025 | 1.09 | 0.75 | | |
| 65- 68 years | -0.01 | 0.023 | -0.47 | 0.75 | | |
| 68 - 73 years | 0.01 | 0.031 | 0.32 | 0.75 | | |
| 73 - 80 years | 0.018 | 0.025 | 0.71 | 0.75 | | |
| **Permutational analysis of variance (PERMANOVA) - Beta diversity** | | | | | | |
|  | **Degree of freedom** | **Sum of squares** | **R^2^** | **Effect size (%)** | **F value** | **P value** |
| **Bray-Curtis (genus level)** |  |  |  |  |  |  |
| 60 - 64 years | 3 | 0.42 | 0.06 |  | 0.85 | 0.14 |
| 65- 68 years | 3 | 0.53 | 0.06 |  | 1.02 | 0.33 |
| 68 - 73 years | 3 | 0.53 | 0.074 |  | 0.912 | 0.019 |
| 73 - 80 years | 3 | 0.42 | 0.04 |  | 0.70 | 0.13 |
| **Bray-Curtis (ASV level)** |  |  |  |  |  |  |
| 60 - 64 years | 3 | 0.71 | 0.058 |  | 0.74 | 0.076 |
| 65- 68 years | 3 | 0.83 | 0.052 |  | 0.84 | 0.049 |
| 68 - 73 years | 3 | 0.80 | 0.065 |  | 0.79 | 0.038 |
| 73 - 80 years | 3 | 0.77 | 0.054 |  | 0.76 | 0.074 |
| **Jaccard distance** |  |  |  |  |  |  |
| 60 - 64 years | 3 | 0.71 | 0.06 |  | 0.77 | 0.55 |
| 65- 68 years | 3 | 0.78 | 0.05 |  | 0.81 | 0.09 |
| 68 - 73 years | 3 | 0.75 | 0.06 |  | 0.77 | 0.14 |
| 73 - 80 years | 3 | 0.77 | 0.056 |  | 0.79 | 0.11 |
| **Weighted UniFrac** |  |  |  |  |  |  |
| 60 - 64 years | 3 | 0.0011 | 0.042 |  | 0.53 | 0.53 |
| 65- 68 years | 3 | 0.00048 | 0.019 |  | 0.30 | 0.78 |
| 68 - 73 years | 3 | 0.00096 | 0.036 |  | 0.42 | 0.11 |
| 73 - 80 years | 3 | 0.0007 | 0.027 |  | 0.37 | 0.95 |
| **Unweighted UniFrac** |  |  |  |  |  |  |
| 60 - 64 years | 3 | 0.65 | 0.062 |  | 0.79 | 0.46 |
| 65- 68 years | 3 | 0.70 | 0.044 |  | 0.71 | 0.81 |
| 68 - 73 years | 3 | 0.74 | 0.064 |  | 0.78 | 0.064 |
| 73 - 80 years | 3 | 0.84 | 0.062 |  | 0.89 | 0.017 |

Alpha diversity was analyzed using ANCOVA for observed richness, Shannon diversity, Faith’s phylogenetic diversity, and Pielou’s evenness, adjusting for baseline values. Beta diversity was evaluated with PERMANOVA at both genus and ASV levels using Bray–Curtis, Jaccard, Weighted UniFrac, and Unweighted UniFrac distances. The table presents estimates, standard errors, test statistics, and adjusted P-values for ANCOVA, as well as degrees of freedom, sum of squares, R^2^, effect size (%), F statistic, and *P*-values for PERMANOVA.

**Supplementary table S9**. Analyses of cognitive performance (Quality Episodic Memory, QEM) at week 24

| **Model/subgroup** | **Term** | **Estimate** | **Standard error** | **T value** | **P value** |
| --- | --- | --- | --- | --- | --- |
| Overall model | (Intercept) | 42.63 | 9.39 | 4.53 | 2.12 ×10^-5^ |
|  | Treatment type (Intervention) | 2.49 | 2.01 | 1.23 | 0.2205 |
|  | QEM baseline | 0.42 | 0.11 | 3.75 | 0.0004 |
|  | Age quartile 65-68 years | 0.97 | 2.80 | 0.34 | 0.72 |
|  | Age quartile 68-73 years | 4.36 | 2.93 | 1.48 | 0.14 |
|  | Age quartile 73-80 years | 5.24 | 2.82 | 1.85 | 0.067 |
|  | Gender (Women) | 3.76 | 1.89 | 1.98 | 0.050 |
|  | Baseline BMI Overweight | -0.10 | 2.54 | -0.04 | 0.96 |
|  | Baseline BMI Obesity | -3.51 | 2.71 | -1.29 | 0.19 |
|  | Age quartile 68-73 years | 4.3695 | 2.9349 | 1.489 | 0.14 |
|  | Age quartile 73-80 years | 5.2474 | 2.8237 | 1.858 | 0.067 |
|  | Gender (Women) | 3.7622 | 1.8926 | 1.988 | 0.050 |
|  | Baseline BMI Overweight | -0.10 | 2.5418 | -0.042 | 0.96 |
|  | Baseline BMI Obesity | -3.51 | 2.71 | -1.29 | 0.19 |
| **Post-hoc contrasts** | Placebo-Intervention | -2.49 | 2.02 | -1.235 | 0.2205 |
| **Model/subgroup** | **Term** | **Estimate** | **Standard error** | **T value** | **P value** |
| Enterotype interaction | (Intercept) | 44.46 | 8.93 | 4.97 | 3.78 × 10^-6^ |
|  | Treatment type (Intervention) | 1.56 | 2.32 | 0.67 | 0.50 |
|  | Baseline enterotype (Enterotype two) | 1.51 | 2.74 | 0.55 | 0.58 |
|  | QEM baseline | 0.42 | 0.11 | 3.64 | 0.00048 |
|  | Gender (Women) | 3.61 | 1.94 | 1.86 | 0.06 |
|  | Treatment type Intervention: Baseline enterotype (enterotype two) | 0.44 | 4.24 | 0.10 | 0.91 |
| **Post-hoc contrasts** | **Enterotype one** Placebo - Intervention | -1.56 | 2.32 | -0.67 | 0.50 |
|  | **Enterotype two:** Placebo - Intervention |  |  |  |  |
| **Model/subgroup** | **Term** | **Estimate** | **Standard error** | **T value** | **P value** |
| BMI interaction | (Intercept) | 48.57 | 9.01 | 5.39 | 7.58 × 10^-7^ |
|  | Treatment type (Intervention) | 1.80 | 4.11 | 0.44 | 0.66 |
|  | Baseline BMI (Overweight) | -0.30 | 3.52 | -0.09 | 0.93 |
|  | Baseline BMI (Obesity) | -2.56 | 3.68 | -0.70 | 0.49 |
|  | QEM baseline | 0.39 | 0.12 | 3.35 | 0.001 |
|  | Gender (Women) | 3.60 | 1.97 | 1.83 | 0.07 |
|  | Treatment type (Intervention: baseline BMI (Overweight) | 0.34 | 5.02 | 0.07 | 0.95 |
|  | Treatment type (Intervention: baseline BMI (Obesity) | -2.61 | 5.52 | -0.47 | 0.64 |
| **Post-hoc contrasts** | **Healthy weight:** Placebo - Intervention | -1.8 | 4.11 | -0.43 | 0.66 |
|  | **Overweight:** Placebo - Intervention | -2.13 | 2.81 | -0.76 | 0.44 |
|  | **Obesity:** Placebo - Intervention | 0.81 | 3.69 | 0.22 | 0.82 |
| **Model/subgroup** | **Term** | **Estimate** | **Standard error** | **T value** | **P value** |
| Age interaction | (Intercept) | 39.15 | 10.12 | 3.87 | 0.0002 |
|  | Treatment type (Intervention) | 4.27 | 4.12 | 1.04 | 0.30 |
|  | Age quartile (65-68 years) | 2.59 | 4.21 | 0.62 | 0.54 |
|  | Age quartile (68-73 years) | 5.22 | 3.82 | 1.36 | 0.18 |
|  | Age quartile (73-80 years) | 5.80 | 3.88 | 1.50 | 0.14 |
|  | QEM baseline | 0.45 | 0.12 | 3.75 | 0.0003 |
|  | Gender (Women) | 3.85 | 1.95 | 1.98 | 0.05 |
|  | Treatment type (Intervention:Age quartile 65-68 years) | -2.76 | 5.49 | -0.50 | 0.62 |
|  | Treatment type (Intervention:Age quartile 68-73 years) | -3.48 | 6.54 | -0.53 | 0.60 |
|  | Treatment type (Intervention:Age quartile 73-80 years) | -0.08 | 5.85 | -0.01 | 0.99 |
| **Post-hoc contrasts** | **60 - 64 years:** Placebo - Intervention | -4.29 | 4.12 | -1.03 | 0.30 |
|  | **65 - 68 years:** Placebo - Intervention | -1.50 | 3.66 | -0.411 | 0.68 |
|  | **68 - 73 years:** Placebo - Intervention | -0.78 | 5.0 | -0.15 | 0.87 |
|  | **73 - 80 years:** Placebo - Intervention | -4.19 | 4.09 | -1.02 | 0.30 |

Linear models (ANCOVA) were used to estimate the effect of anthocyanin intervention on QEM at week 24, adjusting for baseline QEM, age quartile, gender, and baseline BMI. Post-hoc contrasts show estimated differences between placebo and intervention groups within subgroups (enterotype, BMI, age quartile).

**Supplementary Table 10.** Differential associations between gut microbial taxa and Quality of Episodic Memory (QEM) in the intervention study, including overall and age-stratified effects.

| **Genus** | **Estimate** | **Std. Error** | **Statistic** | **P-value** | **Conf. Low** | **Conf. High** | **BH Adjusted P value** | **Analysis type** | **Notes** |
| --- | --- | --- | --- | --- | --- | --- | --- | --- | --- |
| *Acetanaerobacterium* | -6.23 | 1.68 | -3.72 | 0.000396 | -9.58 | -2.89 | 0.00119 | Overall | Intervention-specific effect |
| *Slackia* | 4.58 | 1.35 | 3.39 | 0.00113 | 1.89 | 7.27 | 0.00338 | Overall | Intervention-specific effect |
| *Slackia* | -2.89 | 1.01 | -2.88 | 0.00529 | -4.9 | -0.889 | 0.00793 | Overall | Intervention-independent effect |
| *Phocea* | -2.16 | 0.755 | -2.86 | 0.0055 | -3.67 | -0.656 | 0.0165 | Overall | Intervention-independent effect |
| *Clostridium sensu stricto 1* | -2.37 | 0.845 | -2.8 | 0.0065 | -4.05 | -0.684 | 0.0195 | Overall | Intervention-specific effect |
| *Blautia* | 5.43 | 1.94 | 2.8 | 0.00652 | 1.57 | 9.3 | 0.0196 | Overall | Intervention-specific effect |
| *Clostridium sensu stricto 1* | 1.08 | 0.442 | 2.45 | 0.0168 | 0.201 | 1.96 | 0.0253 | Overall | Intervention-independent effect |
| *Oxalobacter* | -10.4 | 3.83 | -2.7 | 0.00855 | -18 | -2.72 | 0.0256 | Overall | Intervention-specific effect |
| *Harryflintia* | -2.63 | 1.03 | -2.54 | 0.0133 | -4.69 | -0.563 | 0.04 | Overall | Intervention-independent effect |
| *Erysipelatoclostridium* | 2 | 0.804 | 2.49 | 0.0152 | 0.397 | 3.6 | 0.0455 | Overall | Intervention-specific effect |
| **Stratified analysis** | | | | | | | | | |
| *Barnesiella* | 17.4 | 5.94 | 2.93 | 0.0046 | 5.58 | 29.3 | 0.0248 | Stratified | Overall intervention effect |
| *Barnesiella* | -17.7 | 6.82 | -2.59 | 0.0118 | -31.3 | -4.05 | 0.03 | Stratified | Age-specific effect  (65 - 68 years) |
| *Barnesiella* | -15.8 | 6.21 | -2.54 | 0.0133 | -28.2 | -3.39 | 0.03 | Stratified | Age-specific effect  (65 - 73 years) |
| *Barnesiella* | -27.5 | 9.58 | -2.87 | 0.0055 | -46.6 | -8.37 | 0.0248 | Stratified | Age-specific effect  (73 - 80 years) |
| *uncultured Peptococcaceae* | -7.92 | 2.63 | -3.01 | 0.0037 | -13.2 | -2.66 | 0.0334 | Stratified | Age-specific effect (65 - 68 years) |
| *Moryella* | 17.1 | 5.8 | 2.95 | 0.0045 | 5.5 | 28.7 | 0.0343 | Stratified | Overall intervention effect |
| *Moryella* | -16.5 | 6 | -2.75 | 0.0076 | -28.5 | -4.54 | 0.0343 | Stratified | Age-specific effect (65 - 68 years) |
| *Moryella* | -15.7 | 6.04 | -2.6 | 0.0115 | -27.8 | -3.64 | 0.0345 | Stratified | Age-specific effect (73 - 80 years) |

**Supplementary Figures**


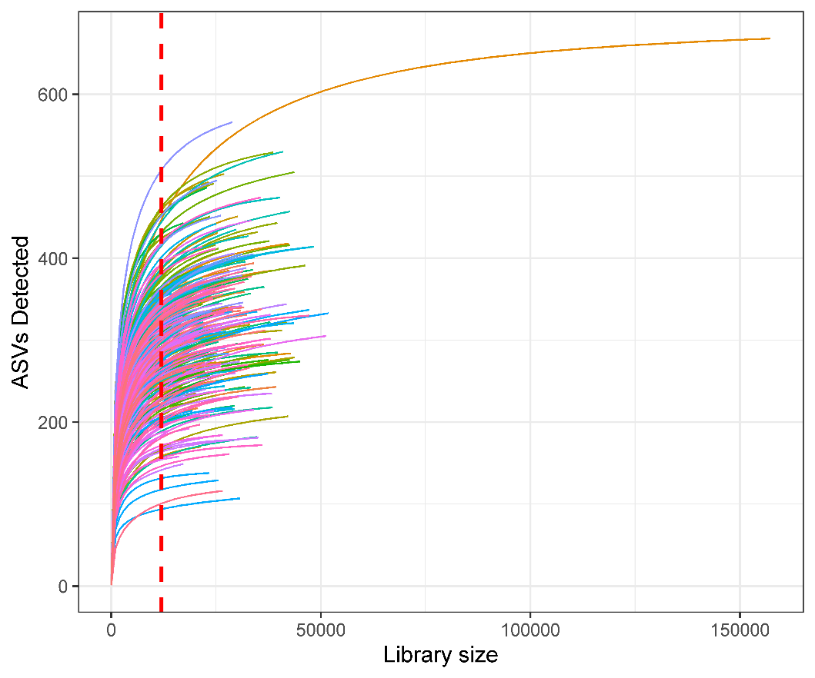


**Supplementary Figure S1.** Rarefaction curves of all samples in the study. The x-axis represents the library size, and the y-axis shows the amplicon sequence variant (ASV) richness at each library size. The red vertical dashed line indicates the optimal rarefaction depth, selected based on the plateau of ASV richness.

| A) | B) |
| --- | --- |
| 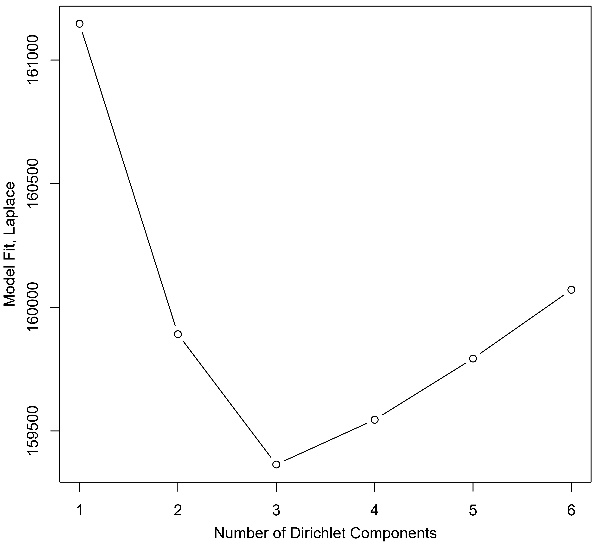 | 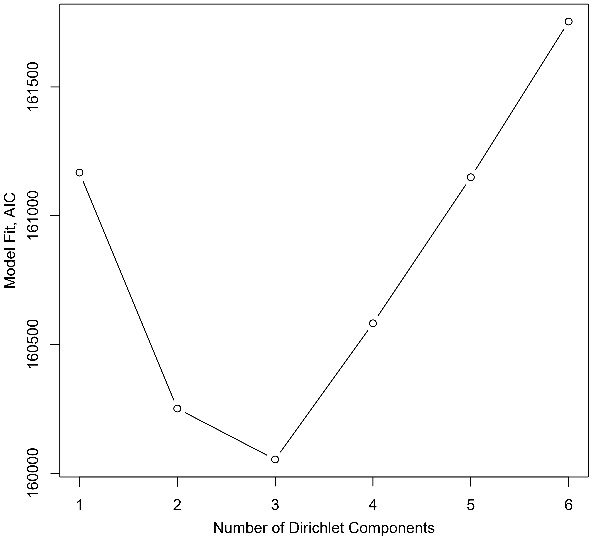 |
| C) | D) |
| 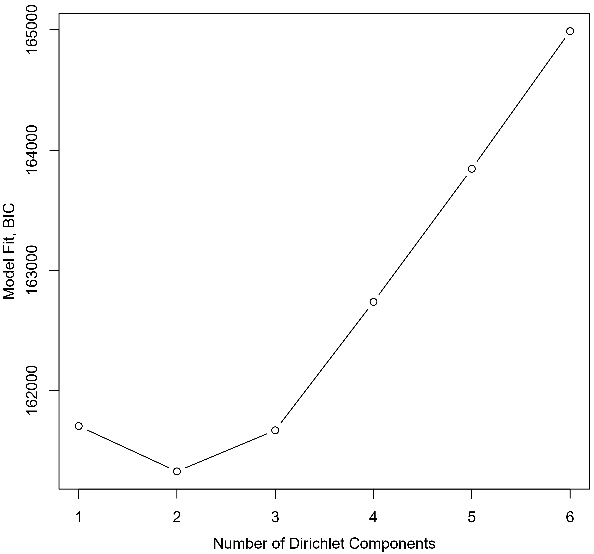 | 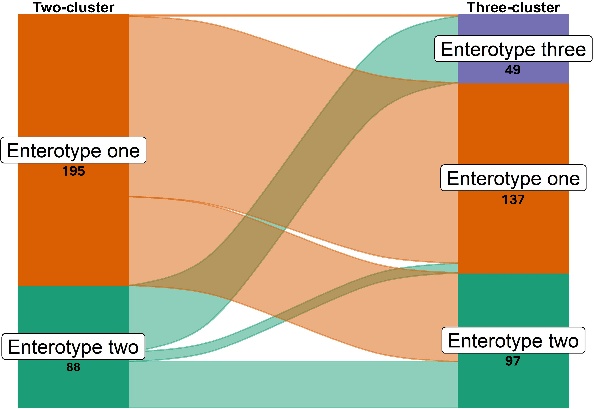 |

**Supplementary Figure S2.** Determination of the optimal number of gut microbial community clusters using the Dirichlet Multinomial Mixture (DMM) model applied to genus-level rarefied count data. Model fit was evaluated using: (A) Laplace approximation, (B) Akaike Information Criterion (AIC), and (C) Bayesian Information Criterion (BIC). The optimal number of components was selected at the “elbow” point in the model-fit curve, where adding more components no longer substantially improves model fit but increases complexity, thus balancing fit and complexity. (D) Alluvial plot comparing clustering results between the two-cluster solution (selected by BIC) and the three-cluster solution (supported by AIC and Laplace). Each flow represents the number of samples shared between enterotypes across models. Stratum heights reflect the relative size of each enterotype, and flow widths indicate sample overlap. The plot illustrates areas of agreement and divergence between clustering solutions, highlighting how individual samples transition between enterotypes.
